# Reimagining pharmacology education

**DOI:** 10.1016/j.pharmr.2026.100126

**Published:** 2026-02-23

**Authors:** Clare Guilding, Roisin Kelly-Laubscher, Margaret Cunningham, Tinne Dilles, David Kennedy, David J. Brinkman, Ali H. Eid, Kelly M. Quesnelle, Ferdi Engels, Simon Maxwell, Arthur Christopoulos, Paul J. White

**Affiliations:** 1 School of Medicine, Faculty of Medical Sciences, Newcastle University, Newcastle upon Tyne, United Kingdom; 2 Department of Pharmacology & Therapeutics, School of Medicine, College of Medicine and Health, University College Cork, Cork, Ireland; 3 Strathclyde Institute of Pharmacy and Biomedical Sciences, University of Strathclyde, Strathclyde, Glasgow, United Kingdom; 4 Department of Nursing Science, University of Antwerp, Antwerp, Belgium; 5 Department of Internal Medicine, Unit Pharmacotherapy and Department of Anesthesiology, Amsterdam University Medical Center, Amsterdam, The Netherlands; 6 Department of Basic Medical Sciences, College of Medicine, QU Health, Doha, Qatar; 7 Department of Biomedical Sciences, Xavier Ochsner College of Medicine, New Orleans, Louisiana, USA; 8 Utrecht University, Utrecht, The Netherlands; 9 University of Edinburgh, Edinburgh, United Kingdom; 10 Drug Discovery Biology, Monash Institute of Pharmaceutical Sciences, Faculty of Pharmacy and Pharmaceutical Sciences, Monash University, Parkville, Victoria, Australia; 11 Australian Research Council Centre for Cryo-Electron Microscopy of Membrane Proteins, Monash Institute of Pharmaceutical Sciences, Monash University, Parkville, Victoria, Australia; 12 Pharmacy and Pharmaceutical Sciences Education, Monash University, Parkville, Victoria, Australia; 13 Faculty of Pharmacy and Pharmaceutical Sciences, Monash University, Parkville, Victoria, Australia

## Abstract

We are arguably experiencing the greatest disruption to higher education in modern history. High-quality education research has demonstrated that active learning and other innovations are significantly more effective than traditional methods. The recent pandemic forced educators to adapt in previously unimaginable ways. Generative artificial intelligence now presents great challenges and opportunities for our approaches to teaching, support of learning and assessment, such as streamlining personalized feedback while raising concerns about academic integrity. This article provides a research informed, expert commentary to support new pharmacology educators in navigating this complex environment. The article is neither a systematic review by design and methodology, nor is it offering comprehensive coverage of the pertinent literature (an insurmountable task, given the breadth of the topic). We highlight how educators in basic and clinical pharmacology are transforming their teaching and curricula to enhance student success in current and future settings. Global initiatives, such as those sponsored by the International Union of Basic and Clinical Pharmacology, including the Pharmacology Education Project and Core Concepts-based curricula, are offering opportunities to enhance pharmacology education by standardizing key concepts, providing open-access learning resources, and fostering international collaboration. These efforts are intended to support alignment of curricula, improve student engagement through interactive materials, facilitating a global exchange of best practices, and supporting educators in adopting innovative teaching methodologies. These initiatives require contributions from pharmacology experts across multiple countries, languages, and cultures. Consequently, this article serves as a call to action to advance innovation and inclusivity in pharmacology education.

**Significance Statement:**

Recent disruptions in higher education have forced educators to adapt in ways that would have previously been unthinkable. The article provides an evidence-based, expert commentary for new pharmacology educators that will assist them to thrive in this complex environment.

## I Introduction

Those individuals around the world teaching pharmacology for the first time have had a bumpy, exciting ride in recent years. Almost every aspect of their teaching role has undergone significant change in the past decades. New pharmacology educators may have less professional identity as a pharmacologist than their predecessors, as their department may have been merged with those from other disciplines, and their content may have been integrated within clinical or biomedical curricula.^1^ Depending on the institution, pharmacology educators may be “education-focused” (ie, with the majority of their workload dedicated to teaching and education-focused scholarship) or may follow a more traditional “Teaching and Research” model (ie, a blend of teaching and laboratory-based or related research activities). They will certainly have encountered student use of generative artificial intelligence (AI),^2^ with all the tremendous opportunities and integrity concerns that it entails with respect to teaching, learning and assessments.^3^ The students that they teach may have a low opinion of textbooks^4^ and may favor gamification or online video repositories.^5^^,^^6^ The mode of teaching in their institution has quite likely changed to incorporate active learning, case-based learning, and/or problem-based learning (PBL). The COVID-19 pandemic profoundly impacted higher education, introducing “drastic” changes to pharmacology education delivery.^7^ Fortunately, pharmacology educational innovation, scholarship, and research has much to offer this passionate new educator.

This article presents perspectives from education leaders in Australia, Europe, Qatar, and the United States, offering commentary on recent changes in the pharmacology education landscape. The authors include basic pharmacological science educators and health professions educators from medicine, nursing and pharmacy. Following agreement on the article’s structure, individual sections were drafted by ≥1 authors with relevant expertise. These drafts underwent multiple rounds of revision and collaborative refinement. The final manuscript was reviewed collectively to ensure alignment with the original intent and coherence across sections.

The paper is structured into 3 key sections: (1) the evolving role of pharmacology educators, exploring historical developments and the interdisciplinary nature of the field; (2) the knowledge, skills, and attitudes that should be taught, focusing on curriculum development and competency-based learning; and (3) research-informed methods for effective teaching, highlighting innovative pedagogical approaches and technological advancements in education. Throughout, we provide practical educator guidance and links to resources, summarizing key insights to support educators in adapting their practices. Although this article includes diverse perspectives from multiple nationalities, we acknowledge that many geographical and national considerations remain beyond its scope.

## II Evolution of the pharmacology educator

### A Evolution of pharmacology as a discipline

All scientific disciplines have a unique history with debatable points of origin, key milestones of discovery, notable periods of decline, and concerns of futureproofing in the era of scientific revolutions and technological advancements.^8^, ^9^, ^10^ Pharmacology as a scientific discipline has a rich history rooted in humanity’s understanding and manipulation of natural compounds for therapeutic purposes. Early healers and practitioners relied heavily on plants, minerals, and animal-derived compounds to treat illnesses, as documented in classic texts such as Dioscorides’ *De materia medica* (∼ad 50–70), which detailed around 600 substances to treat over 1500 illnesses. Such early therapeutic frameworks were based largely on observation and entrenched beliefs, such as humorism and the doctrine of signatures,^11^ passed down from teacher to pupil and rarely questioned for centuries. Although some remedies in early pharmacopoeia proved effective, such as willow bark and opium for managing fever and pain, many were ineffective or even harmful. Over time, skepticism grew among physicians in human and veterinary medicine regarding the reliability of this inherited body of knowledge.^11^^,^^12^

The transition from traditional medicine to a more systematic and scientific approach began during the Renaissance, driven by pioneers such as Paracelsus. He rejected Greek humorism, asserting that diseases had specific causes that could be treated with chemical-based remedies,^12^ and highlighting the importance of dose-response relationships. Advancements in methods of chemical isolation, alongside the emergence of modern organic-/bio-chemistry and experimental physiology paved the way for investigating the physiological effects of individual chemicals and identifying their specific sites of action. As a result, pharmacology evolved from an empirical branch of medicine to a science focused on understanding the precise mechanisms of drug action.

Pharmacology is inherently interdisciplinary, as it combines and integrates several academic disciplines such as chemistry, physiology, and even mathematics, each with their own terminology, theoretical frameworks, and methodological traditions. Although modern pharmacology emerged from advances in biochemistry, physiology, and pathophysiology, it has also profoundly influenced these fields by introducing new methods for analyzing life processes and treating diseases. As early as 1704, pharmacology was described as a “boundless field.”^13^ However, its very expansiveness places a challenge on the pharmacologist to define the discipline, and on the pharmacology educator to teach and prioritize the core curriculum.^14^ Rudolf Buchheim (1820–1879) was one of the first Professors of Pharmacology and among the earliest educators to establish pharmacology as a distinct scientific discipline within the medical curriculum.^15^ He championed 2 core principles for teaching pharmacology: first, that drugs should be grouped by how they act rather than by their source or chemical structure, and second, that these mechanisms must be elucidated through scientific inquiry to support rational therapeutic use. By shifting teaching in his institution away from a reliance on *materia medica* and embedding this experimental approach into undergraduate training, he laid essential groundwork for modern pharmacology education.

As pharmacology developed into a thriving scientific discipline, universities saw a proliferation of pharmacology departments and the establishment of named chairs in the field.^16^ However, this growth also coincided with an increasing division between basic/fundamental science and clinical departments. Early pharmacologists were often physicians, as reflected in the British Pharmacological Society (BPS), where 90% of members at its founding in 1931 had medical training.^11^^,^^17^ During the early to mid-20th century, it was common for medical students in the United States and United Kingdom to take 1 or 2 years after their basic training to conduct research, including pharmacological studies, before either returning to clinical practice or continuing as scientists.^18^ These clinically trained pharmacologists often became instructors for both science and medical students.^19^ The introduction of residency programs for junior doctors, primarily within clinical rather than scientific departments, reduced the number of clinicians engaged in basic pharmacological research.^20^ By 1964, the proportion of medically trained pharmacologists in the BPS had declined to 25%.^11^ Within universities, this division had a notable impact. Basic science departments, including pharmacology, focused on supporting teaching and conducting increasingly complex research that required specialized expertise. In contrast, clinical departments prioritized service delivery. This separation led to a divergence in how pharmacology was taught. In healthcare programs, basic pharmacology often emphasized research topics with limited relevance to clinical practice, whereas the practical application of medicines was reserved for later stages of training. A 1962 talk at Yale University on the contribution of basic science to the future of medical education noted these changes:

Some of the startling changes can be surmised from a quick glance at the schedule of seminars held by the basic science departments of the Yale Medical School. A random sample for the week of February 12 reveals a Pharmacology seminar on “the interaction of anomalous nucleotides with polynucleotide phosphorylase,” a Biochemistry seminar on “the assembly of the hemoglobin molecule,” a Microbiology seminar on ‘in vitro hybridization and synthesis of alkaline phosphatase,” and so on. The subject matter may seem foreign and the relevance to medicine may seem remote.^20^

This growing disconnect was reflected in the continued decline of medically-trained pharmacologists, with their proportion in the BPS falling to 14% by 1971. In 1976, Csaky, marking the 100-year anniversary of Rudolf Buchheim’s seminal work on pharmacology education, raised concerns about an “identity crisis” in medical school pharmacology.^21^ The paper highlighted the gap between the foundational scientific knowledge of pharmacology acquired by researchers and its practical application in patient care. Csaky argued that clinicians needed more than rote memorization of which drugs to prescribe; they required a deeper understanding of pharmacological mechanisms to adapt to the rapidly evolving field of drug development. Within higher education, he emphasized the importance of providing medical students with a solid foundation in the scientific principles of pharmacology to prepare them for applying this knowledge to emerging therapeutics.

#### 1 Pharmacology as an interdisciplinary subject

Pharmacology education has evolved significantly since the late 1970s, both in terms of what is taught and how it is delivered. Today, we are moving into an interdisciplinary era that emphasizes connections across foundational sciences and healthcare professions, and bridges the divide between preclinical and clinical education, reshaping how pharmacology is taught. This shift has been driven by a range of complex factors including changing regulatory requirements (see section Evidence-Based Approaches to Determining What We Teach and What Students Need to Learn), advancements in our understanding of effective pedagogical approaches (see section How We Teach), advancements in therapeutics, and evolving departmental structures. There has been a concerted effort to connect pharmacology education at the undergraduate level to real-world applications, whether in drug design and development, or the clinical use of medicines. In healthcare education, the trend has been toward integrating basic and clinical medicine within integrated curricula (see section Evidence-Based Approaches to Determining What We Teach and What Students Need to Learn). This is complemented by the introduction of industrial placements in pharmacology programs, and redesigned curricula that emphasize active learning and authentic assessment methods (see section How We Teach).

The trend toward greater interdisciplinarity and the integration of pharmacology into broader disciplines is marked by the merging and loss of named pharmacology departments over past decades. Table 1 highlights this trend by comparing the names of UK departments that explicitly include “Pharmacology” from Griesbacher’s 2003 list of “Pharmacology Departments Worldwide,”^22^ with the names of departments at the same universities in 2025. This shows a 74% reduction in the number of UK departments explicitly named “Pharmacology” from 2003 to 2025. Similar reductions are seen in several countries, illustrated by the examples in Table 2.

**Table 1:** Name changes of UK pharmacology departments from 2003 to 2025

2003 Name (Griesbacher^22^)	2025 Name^a^
Department of Medicine and Therapeutics, University of Aberdeen	Institute of Medical Sciences, University of Aberdeen
Department of Pharmacy and Pharmacology, University of Bath	Department of Life Sciences, University of Bath
Department of Therapeutics and Pharmacology, Queen’s University	School of Pharmacy, Queen’s University Belfast
Department of Pharmacology, University of Birmingham	Institute of Clinical Sciences, University of Birmingham
Medicines Research Unit, Aston University	School of Biosciences, Aston University
Molecular Biosciences Research Group, Aston University	School of Biosciences, Aston University
Department of Pharmacology, University of Bristol	School of Physiology, Pharmacology, and Neuroscience, University of Bristol
Department of Pharmacology, University of Cambridge	Department of Pharmacology, University of Cambridge
Department of Pharmacology Therapeutics and Toxicology College of Medicine, University of Wales	School of Medicine, Cardiff University
Division of Pharmacology, Welsh School of Pharmacy University of Wales	School of Pharmacy and Pharmaceutical Sciences, Cardiff University
Department of Pharmacology and Neuroscience, University of Dundee	School of Life Sciences, University of Dundee
Interdisciplinary Research Group of Neuropharmacology, Division of Neuroscience University of Edinburgh	Centre for Discovery Brain Sciences, University of Edinburgh
School of Pharmacology, University of Glasgow	School of Infection and Immunity, University of Glasgow
Division of Neuroscience and Biomedical Systems Institute of Biomedical and Life Sciences, University of Glasgow	Institute of Infection, Immunity and Inflammation, University of Glasgow
Molecular Pharmacology Group Division of Biochemistry and Molecular Biology Institute of Biomedical and Life Sciences, University of Glasgow	Institute of Molecular, Cell and Systems Biology, University of Glasgow
Division of Pharmacology Department of Veterinary Preclinical Studies, University of Glasgow	School of Veterinary Medicine, University of Glasgow
Department of Physiology and Pharmacology School of Pharmacy University of Strathclyde	Strathclyde Institute of Pharmacy and Biomedical Sciences (SIPBS), University of Strathclyde
Department of Cell Physiology and Pharmacology, University of Leicester	School of Biological Sciences, University of Leicester
Department of Pharmacology and Therapeutics, University of Liverpool	Department of Pharmacology, University of Liverpool
Division of Pharmacology and Therapeutics King’s College, University of London	School of Cancer and Pharmaceutical Sciences, King’s College London
Sackler Institute of Pulmonary Pharmacology, King’s College University of London	School of Cancer and Pharmaceutical Sciences, King’s College London
Section of Behavioural Pharmacology, Institute of Psychiatry, King’s College University of London	Institute of Psychiatry, Psychology and Neuroscience, King’s College London
Department of Clinical Pharmacology, Cardiovascular Science National Heart and Lung Institute, Imperial College London	Department of Metabolism, Digestion and Reproduction, Imperial College London
Department of Pharmacology Queen Mary and Westfield College University of London	School of Biological and Behavioural Sciences, Queen Mary University of London
Department of Biochemical Pharmacology, Queen Mary and Westfield College University of London	School of Biological and Behavioural Sciences, Queen Mary University of London
Department of Pharmacology, University College London	UCL Division of Biosciences, University College London
Centre for Clinical Pharmacology Therapeutics and Toxicology, University College London	UCL Division of Medicine, University College London
Department of Life Sciences, University of East London	School of Health, Sport and Bioscience, University of East London
Department of Pharmacology, Royal Free Hospital of Medicine	UCL Medical School, University College London
Department of Neuroendocrinology (previously Department of Pharmacology), Charing Cross and Westminster Medical School	Faculty of Medicine, Imperial College London
Department of Clinical Pharmacology and Therapeutics, Charing Cross and Westminster Medical School	Faculty of Medicine, Imperial College London
School of Biological Sciences, University of Manchester	Faculty of Biology, Medicine and Health, University of Manchester
Department of Pharmacological Sciences, Newcastle University	Biosciences Institute, Newcastle University
Psychopharmacology Section, Division of Psychiatry, School of Community Health Sciences, University of Nottingham	School of Life Sciences, University of Nottingham
Department of Pharmacology, University of Oxford	Department of Pharmacology, University of Oxford
Department of Clinical Pharmacology, University of Oxford	Department of Pharmacology, University of Oxford
Anatomical Neuropharmacology Unit Medical Research Council	Medical Research Council Brain Network Dynamics Unit, University of Oxford
Section of Pharmacology, School of Pharmacy and Biomedical Sciences, University of Portsmouth	School of Pharmacy and Biomedical Sciences, University of Portsmouth

Departments are ordered alphabetically according to city using reference data from Griesbacher.^22^

a For institutions where specific pharmacology departments were merged or renamed, the current broader departments or schools are listed where pharmacology or related subjects are now housed. Some institutions have integrated pharmacology into wider interdisciplinary institutes or schools, reflecting the trend toward multidisciplinary education and research.

**Table 2:** Number of departments retaining the explicit mention of “pharmacology” in their departmental title from 2003 to 2025 across a range of countries

Region	2003	2025	Percent Decrease
Australia	9	5	44
Belgium	14	5	64
Canada	14	9	36
South Africa	12	4	67
United Kingdom	34	9	74

Using the dataset from Griesbacher.^22^

The movement toward interdisciplinary departments has been prompted by both structural and educational factors, largely reflecting the increasingly collaborative nature of modern science. Innovations in drug development, biomedical research, and personalized medicine rely on a combined understanding of a myriad of subjects including pharmacology, cell biology, genetics, chemistry, and clinical sciences. Interdisciplinary research combining knowledge from multiple fields can address complex problems beyond the scope of a single discipline.^23^^,^^24^ Indeed, blending of disciplines has catalyzed the rise of emerging areas such as bioinformatics, systems biology, and personalized medicine.^25^ Such cross-disciplinary research can be facilitated through the merging of departments and encouragement of collaborative grants and research projects. There is an additional economic component to the merging of departments, where financial pressures at many universities can drive efforts to reduce administrative costs and optimize the use of research infrastructure.

From an education perspective, the focus on translating foundational research into clinical practice underpins the need for curricula spanning molecular mechanisms, therapeutic strategies, and patient outcomes.^26^ In healthcare education, the shift toward competency-based education, where students must demonstrate proficiency across integrated knowledge areas, has prompted the development of programs that embed pharmacology within a broader interdisciplinary framework. The aim is to emphasize the interconnectedness of subjects such as physiology, pathophysiology, biochemistry, communication and professionalism in understanding mechanisms of disease, therapeutic approaches, and patient care^16^^,^^27^, ^28^, ^29^, ^30^ (see section Evidence-Based Approaches to Determining What We Teach and What Students Need to Learn). The rationale for teaching pharmacology alongside other disciplines is further supported by new education models that promote active learning and problem-solving skills, which are better supported by integrated courses that encourage the application of knowledge across disciplines^16^^,^^29^^,^^30^ (see section How We Teach).

The loss of pharmacology departments through their merger into larger multidisciplinary units, while offering numerous advantages outlined above, also presents challenges for education. Integration of multiple disciplines risks the development of overloaded curricula, where students struggle to meet the diverse demands of several subjects (see section Evidence-Based Approaches to Determining What We Teach and What Students Need to Learn). Such curricula may compromise the comprehensiveness of pharmacology education, resulting in graduates who lack essential knowledge and skills specific to the field. Faculty may need to teach across broader subject areas, and pharmacology could be delivered by nonspecialists, potentially affecting the quality and rigor of pharmacology education and research. More broadly, the absorption of smaller disciplines into larger academic units risks diminishing their visibility, weakening their representation in decision-making, and making it more difficult to secure targeted funding or attract students with a clear interest in the field. The loss of autonomous departments may also hinder recruitment, curriculum innovation, and the long-term sustainability of specialist career paths.^31^

Looking ahead, the ongoing trend toward institutional mergers at the level of entire higher education institutions (HEIs) could amplify these challenges. Such large-scale restructuring often leads to the reorganization or closure of programs seen as financially marginal or misaligned with new strategic goals. In this context, smaller or more specialized disciplines, particularly those without strong cross-subsidy from larger enrolments, may come under increasing pressure. The implications of HEI mergers therefore extend beyond administrative efficiency; they may reshape the academic landscape in ways that challenge the sustainability of certain fields, potentially accelerating trends already set in motion by earlier departmental consolidations.^32^

#### 2 The role of pharmacology in modern healthcare and society

Pharmacology and the broader pharmaceutical sciences underpin one of the fastest growing sectors of the global economy, driven by rapid advances in therapeutic design and postgenomic technologies.^33^^,^^34^ Research and development pipelines in the pharmaceutical industry are expanding at an unprecedented rate, spurred by innovations in biologics, genomics, and precision medicine.^35^ Concurrently, medicines continue to occupy an ever-larger share of healthcare practice and spending, delivering significant improvements in health outcomes but also adding complexity to clinical decision-making. In the United States, spending on retail prescription drugs increased by 11.4% between 2022 and 2023, to $449.7 billion, accounting for 1.62% of Gross Domestic Product.^36^ In the UK health sector, spending on medicines typically makes up 20% of all nonwage costs (around £20 billion) and this proportion continues to rise.^37^^,^^38^ In Belgium, retail pharmaceutical expenditure amounts to €2.92 billion and is expected to rise by 23.5% between 2022 and 2027.^39^ Belgian hospital outpatient pharmaceutical expenditures amount to €2.86 billion, with an expected increase in the same period of 78.4%.^39^ In Europe, the pharmaceutical sector has shown substantial growth, with research and development spending increasing by an average of 4.4% annually between 2010 and 2022, from €27.8 billion to €46.2 billion, contributing a total of €311 billion to the EU-27 economy and directly employing over 865,000 people in 2022.^40^ In Europe, the Organisation for Economic Cooperation and Development projects a 48.0% increase in total pharmaceutical expenditures between 2022 and 2027.^41^

At the same time, public expectations of the safety and efficacy of medicines have never been higher. Unfortunately, patients’ safety is being compromised by an increase in adverse effects of drugs and prescribing errors, many of which are avoidable.^42^ One in 20 patients experiences preventable medication-related harm. One fourth of the harm is severe or potentially life-threatening. The prevalence is relatively higher in the most vulnerable patients, for example in low- and middle-income countries or geriatric care units.^43^ Against that background, the need to provide effective education in pharmacological sciences for researchers, clinicians, and arguably for the public, has never been greater.^44^ These individuals will play important roles in drug development, clinical trials, regulation, appraisal, prescribing and supervision of therapy, and will have to do so with regard to a science that is changing ever more rapidly.

### B Pharmacology students: A growing and diverse cohort

Pharmacology education today includes students in a broad range of academic programs, such as medicine, pharmacy, nursing, dentistry, veterinary science, and biomedical sciences (see “Pharmacology training programs” below). Each program will have distinct entry criteria, thus students enter with varied prior learning experiences and progress toward different objectives, such as prescribing competence, pharmaceutical care, or research literacy. Such diversity influences students' learning approaches and how pharmacology curricula are designed, as educators aim to balance relevance and depth without overloading programs.^45^ This becomes increasingly difficult when students from different programs are cotaught. Recognizing diversity in the cohorts we teach is essential to ensure that each qualification achieves the level of pharmacological understanding needed for its professional context. Beyond these programmatic considerations, broader demographic shifts in higher education have further diversified pharmacology cohorts, influenced by globalization, policy reforms, and enhanced accessibility. These shifts are evidenced in the increasingly diverse profiles of pharmacology students, who represent a broad spectrum of socioeconomic backgrounds, cultural identities and academic experiences.

The globalization of higher education has led to an unprecedented increase in students enrolling at HEIs in countries different to their nationality, that is, international students. According to data from Organisation for Economic Cooperation and Development, international students represented approximately 10% of the global higher education population in 2020, with this figure rising to as much as 26% in United Kingdom and 48% in Luxembourg.^46^ More students travel for education, facilitated by improved transportation but also coordinated changes among countries. For example, changes implemented through the Bologna Process make it easier for students to travel and get experience of different countries and cultures, enjoy modules that might not be available in their own country and allow for greater postgraduate employability. This flexibility has allowed countries with excess program places to address shortages elsewhere and is also of benefit to educators.^47^ These changes allow lecturers to teach similar courses in different countries and collaborate on offerings with other institutions. High-demand courses such as medicine and veterinary science are particularly affected, as some countries cannot accommodate all the students seeking to pursue these careers, despite the need for more professionals. For example, many Irish students are enrolled in undergraduate degrees in medicine and veterinary science in Poland, and German students are enrolled in medical degrees in Austria or Hungary.^48^, ^49^, ^50^

**Fig. 1:**
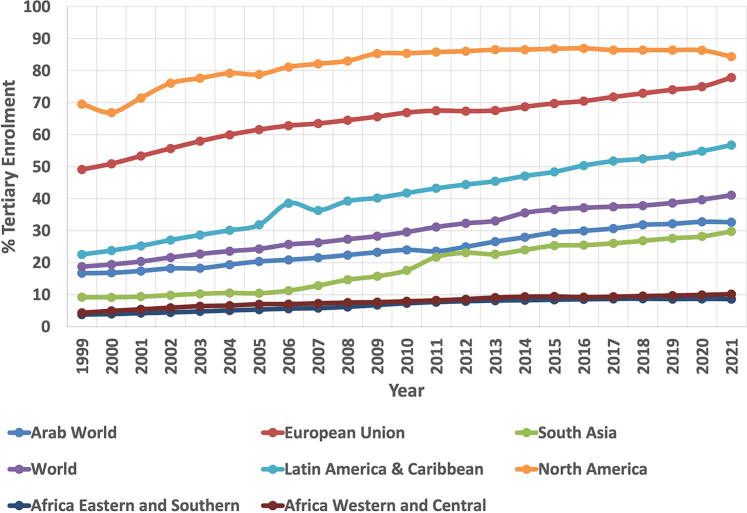
Enrolment in tertiary education across the world. Data is presented as a percentage of the population of the age group that is enrolled in tertiary education. Results may be influenced by proportion of the population within that age group and reliability of birth registries in each country. The figure was created using the World Bank Group databank^57^ and enhanced for clarity using Inkscape.

Pharmacology programs, particularly those within prestigious universities, attract international students from regions with limited access to advanced pharmaceutical sciences. Many universities even offer branch campuses for the provision of pharmacy programs to international students.^51^ This internationalization enriches the pharmacology classroom by fostering diverse perspectives, promoting cross-cultural collaboration, and broadening the scope of academic discourse. The growing diversity within the student body is also increasingly apparent among domestic students, as higher education initiatives to improve accessibility have welcomed individuals from a wider range of underrepresented socioeconomic, ethnic, age, and disability backgrounds.^52^, ^53^, ^54^ In the United Kingdom, almost 40% of young people now enter higher education, a significant rise compared with previous decades.^55^ In the Netherlands over the last decade, more Dutch people have been attending higher vocational education or university. In 2013, 28% of people aged 15–74 years had an higher vocational education or university diploma, but in 2023 that share was 36%.^56^ Figure 1^57^ shows the percentage of the population enrolled in tertiary education across the world.

Pharmacology is recognized as a degree that offers students from poor socio-economic backgrounds a high chance of social mobility.^58^ An increasing enrolment of mature students, often pursuing career changes or advanced specializations, adds another layer of diversity to classrooms. These students bring valuable professional experience but often balance their studies with significant personal and professional responsibilities, such as careers, family commitments, and other obligations outside of university life. The increasing diversity of cohorts highlights the importance of flexible learning options, such as online resources and blended teaching methods, alongside inclusive practices to address disparities in academic preparation and support students navigating financial pressures or competing demands beyond academia. The rise in international students likewise presents challenges for educators. Variations in prior academic preparation and language proficiency may require the implementation of tailored teaching strategies to ensure equitable learning outcomes for all.^59^^,^^60^

#### 1 Pharmacology training programs and employment

Students engage with pharmacology through degree programs and professional development courses, including undergraduate and graduate programs spanning healthcare, basic and clinical research, and drug development. All healthcare professionals—doctors, pharmacists, nurses, physician associates, dentists, vets, and others, increasingly require a solid grounding in pharmacology. Beyond the clinic, pharmacological research fuels the discovery of new drugs and therapeutic innovations. At the undergraduate level, standalone pharmacology degrees provide students with comprehensive foundational knowledge in drug actions, pharmacokinetics, toxicology, and experimental techniques. Graduates from these programs progress onto a range of different careers, including in the pharmaceutical industry or academia. These were the 2 major employment outcomes for pharmacology graduates noted in a 2017 report commissioned by the BPS.^61^ The report analyzed undergraduate and postgraduate education, work experience opportunities and employment patterns for pharmacologists in the United Kingdom. The number of pharmacology undergraduates in the United Kingdom increased by 40% between 2007 and 2015, with an average of 6.4 applications per first-year place, reflecting strong demand. Pharmacology’s growth rate surpassed other medical sciences, and the number of graduates exceeded first-year entrants, suggesting students often transferred into the field. The number of postgraduate students in pharmacology, pharmacy, and toxicology courses grew by 6% a year since 2003, again, much faster than the average across all subjects (3.1%). In 2015, 28 universities in the United Kingdom offered undergraduate pharmacology degrees under 17 titles, highlighting variability in program definitions.^61^ The report noted that traditional “pharmacology” degrees were declining, whereas “medical pharmacology” and “applied pharmacology” were rising. Whether these changes reflected substantive differences in program content versus branding to emphasize career relevance was unclear. Overall, the data showed strong interest and growing enrolment in pharmacology programs in the United Kingdom, with the discipline increasingly featured in allied degrees.

Modern pharmacology is taught at varying depths across the diverse courses it is found within, as highlighted by a study that evaluated pharmacology curricula in Australian science and health-related degree programs.^62^ Using a national survey across 22 institutions and 147 courses, it found that pharmacology content was broadly consistent across degree programs, but the breadth and depth of coverage varied. Pharmacy programs devoted the most lecture hours, whereas medicine focused on integrated teaching approaches (see section How We Teach). Science programs emphasized standalone courses, and nursing programs had the least comprehensive coverage of pharmacology topics.^62^ Nurses are generally trained in basic pharmacokinetics and pharmacodynamics, although a study across 14 European countries found only 70% of students evaluated the training as sufficient.^63^ More fundamental topics such as medicinal chemistry and toxicology were seldom or never addressed, with the focus more on pharmaceutical care.^63^ Evolving roles such as nonmedical prescribing authority for nurses, are expanding worldwide and requires more in-depth pharmacological understanding.

Sometimes, the challenge is not deciding what to teach, but rather how to integrate it into an already packed program that must also address multiple other disciplines, skills, competencies, and graduate attributes. Over the past few decades, clinical pharmacology has been integrated horizontally and/or vertically across most medical curricula, which has reduced dedicated teaching hours and made the subject less visible, at times even causing it to disappear entirely.^26^ Indeed, very few teaching hours in European medical curricula are devoted to clinical pharmacology education (median 68 hours; ± 2%–3% of total study load).^64^ Trends are similar in the United States where clinical pharmacology education averages 109 hours.^27^ The study load for clinical pharmacology tends to be lighter compared with other subjects, particularly those focused on diagnostic subjects. Curriculum designers often prioritize diagnostic subjects over therapeutic subjects, as the former is generally viewed as more challenging.^65^

Appeals to preserve pharmacology as a distinct discipline have been made globally over the years.^66^ Researchers who aimed to map pharmacology or pharmaceutical care in education encountered difficulties as coordinators of educational programs were not able to provide overviews. The integration in a broad range of courses throughout the curriculum, often resulted in a more scattered, and less controlled program content.^63^ In 2006, the clinical pharmacologist and later UK Government Chief Scientific Adviser (2018–2023) argued “teaching and training across the range of areas of pharmacology need the discipline to exist as a discrete entity and require, above all, role models to inspire the next generation.”^67^ Fast-forward to almost 20 years later and the educator’s sentiment of preserving the discipline of pharmacology has evolved. One of the major considerations now relates to the practicalities of how to teach pharmacology effectively with the rapid expansion of knowledge and skills that graduates must possess.^16^^,^^68^

### C Pharmacology educators: Balancing research, teaching, and pedagogy

Who then will be the (future) teachers of pharmacology and clinical pharmacology able to train and inspire the next generation of students? Analysis of job adverts for lecturers on undergraduate pharmacology degrees reveals that the majority require applicants to have a PhD in pharmacology or a related discipline. Pharmacology is frequently taught within interdisciplinary biomedical science departments, where educators may also teach related subjects such as physiology, anatomy, pathology, or microbiology.^69^ This diversity can enrich pharmacology education by bringing multiple perspectives, but it may also create structural challenges for curriculum design and alignment, particularly when educators’ primary academic training lies outside pharmacology. Teaching outside one’s discipline can be challenging, especially for staff who are new to teaching in general. Based on our observations across many institutions around the world, while the structure of teaching roles varies substantially, pharmacology educators commonly fall into 2 broad categories: those in predominantly education-focused roles and those who combine education with clinical or laboratory research. Within pharmacology education for healthcare degrees, we find basic science educators, who often teach the foundational and early years’ education, and clinical educators, for example practicing physicians, pharmacists, or vets, who teach the more clinically applied pharmacology, though some also cover foundational pharmacology.^70^

In the past, pharmacology educators were predominantly researchers or clinicians, with teaching often being a secondary responsibility.^71^ Similar to colleagues in other fields, they lacked formal training in pedagogy and therefore relied on traditional methods, such as lectures, which often did not emphasize critical thinking or the practical application of knowledge. However, as higher education has progressed, the value of high-quality teaching has become increasingly apparent.^72^ The modern pharmacology educator operates at the intersection of scientific research, education, and pedagogical innovation, and increasingly they balance research (scientific or education) with high-quality teaching, leadership, and administration responsibilities. As with educators in most disciplines, pharmacology educators must draw on an integrated set of knowledge, skills, and professional attributes to enable their students to succeed.^68^ Moreover, teaching the more clinically applied pharmacology requires lecturers to understand the roles and responsibilities that the students will take up after graduation, so they can select and teach the most relevant course content.

A fundamental requirement of the pharmacology educator is expertise in core disciplinary knowledge: for example, pharmacokinetics, pharmacodynamics, drug interactions, toxicology, and therapeutics. Educators must further have knowledge and insights in the fields of chemistry, biochemistry, cell biology, physiology, and pathophysiology (among others), and be able to integrate these disciplinary fields. The relative importance of these disciplines naturally depends on the educator’s field of work, that is, involvement in health sciences-oriented curricula or curricula focused more on drug development.

Building on this foundation of core disciplinary knowledge, pedagogical content knowledge (PCK) plays a crucial role in effective pharmacology education as in other disciplines, bridging subject mastery with teaching methodologies to create meaningful learning experiences. PCK encompasses an educator’s ability to understand how students learn specific topics, identify common misconceptions, and employ instructional strategies that facilitate comprehension and retention.^73^ Great educators need both general pedagogical skills, which focus on classroom management and instructional delivery, and PCK, with which educators anticipate subject-specific student challenges and adapt their teaching accordingly.^74^

Pharmacology educators develop PCK through ongoing engagement with their discipline, which may involve research encompassing both the Scholarship of Teaching and Learning (SoTL) and Discipline-Based Education Research (DBER), 2 complementary approaches to investigating and improving student learning.^75^^,^^76^ SoTL aims to advance instructional practice through the systematic study of teaching and/or learning, and the public sharing and review of such work.^77^ It is highly contextualized, often driven by individual academics seeking to refine their teaching strategies through reflective practice, classroom-based research, and collaborative discussions. Although pharmacology educators are trained in experimental methods common to the life sciences, SoTL often relies on methodologies rooted in the social sciences, requiring educators to engage with new research paradigms. Engagement in SoTL can begin at a small scale through faculty development programs, pedagogical workshops, and reflective teaching practices, eventually extending to more formal qualifications in Education at a postgraduate level. These opportunities help educators build expertise in pedagogical research while equipping them with tools to enhance student learning in pharmacology.

In contrast, DBER applies systematic research methodologies to examine how students learn within a particular discipline. DBER in pharmacology is embedded in the discipline’s knowledge base, aligning research priorities with the specific challenges of teaching, for example, drug mechanisms, pharmacokinetics, and therapeutic applications. Unlike SoTL, which often emphasizes educator experience and classroom practice, DBER seeks generalizable insights that inform curriculum design, assessment strategies, and student engagement across different educational contexts. It is influenced by cognitive science, educational psychology, and broader science education research, often investigating how students conceptualize complex pharmacological processes.^78^, ^79^, ^80^ Pharmacology DBER benefits from national and institutional collaborations, supporting large-scale studies that can impact teaching beyond individual classrooms. Despite their differences, SoTL and DBER are not mutually exclusive, and many pharmacology educators engage in both, using SoTL to refine their teaching approaches in specific courses and DBER to generate broader insights applicable across institutions. Publishing research on pharmacology education, presenting at discipline-specific teaching conferences, and collaborating with interdisciplinary education researchers can provide valuable professional development opportunities,^23^ and is an increasingly recognized part of promotion and tenure programs for educators.^81^

**Fig. 2:**
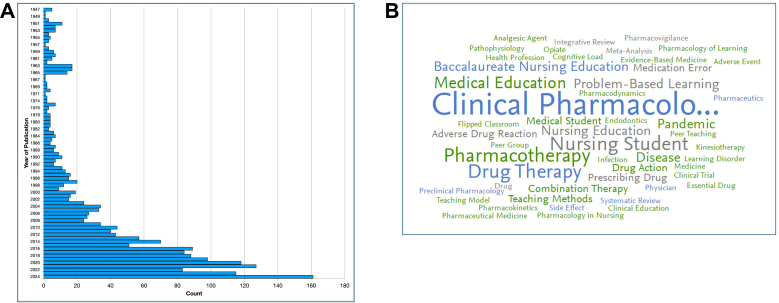
An illustrative representation of the rise in pharmacology education research. (a) Data were visualized from PubMed based on the scholarly output associated with pharmacology education research between 1947 and 2025. Simple Pharmacology AND Education search terms were applied to search publication titles and abstracts, with 216 scholarship papers retrieved. (b) Word cloud created in SciVal from the following search terms: (“Pharmacology education” OR “Pharmacology teaching” OR “Pharmacology educator” OR “Teaching pharmacology” OR “Learning pharmacology” OR “Pharmacology classroom” OR “Pharmacology undergraduate”). Applied filters: NOT Neuroscience AND NOT Engineering, Publication year range: 2019-present.

The range of topics and rise in pharmacology education research over the last 78 years is illustrated in Fig. 2. Although the results presented do not reflect a comprehensive search, they were selected to demonstrate the increasing visibility and diversification of pharmacology education, particularly in the postpandemic era. The earliest paper retrieved (1947) “Suggestions for new objectives in pharmaceutical education” and the most recent (2025) “AI in action: Changes to student perceptions when using generative artificial intelligence for the creation of a multimedia project-based assessment,” both address contemporary challenges in their respective contexts. However, the nature of these challenges has evolved considerably. Although foundational concerns around curriculum and professional identity remain, the complexity and speed of change, particularly driven by technological advances, now pose significant demands on educators. Overall, the search revealed that pharmacology education within medical and nursing programs was most represented across the retrieved papers. All titles had a teaching focus; however, a more robust evaluation of pharmacology education research would require a comprehensive literature search using a wider set of databases beyond PubMed.

In addition to pedagogical research skills, lecturers require diverse technical and organizational skills. They must design and structure curricula, manage courses across different formats, deliver engaging lessons, supervise research, assess students in meaningful ways, and ensure curricula meet the requirements of Accrediting and Regulatory bodies. The rapid advancement of technology has transformed pharmacology education; thus, educators must have the technological skills to utilize and adapt to such advancements. Digital learning platforms, AI-driven tools, and virtual and augmented reality simulations create immersive and interactive learning environments.^16^^,^^29^ Although technology provides opportunities to enhance student engagement and personalize learning experiences, it is the role of the educator to critically assess its pedagogical value. Effective technology integration involves balancing digital innovation with evidence-based teaching practices and engaging in continued professional development to ensure that the digital tools are used effectively and confidently.

Beyond knowledge and technical skills, educationalists propose that a modern university educator must demonstrate professionalism through enthusiasm, authenticity, and continuous self-improvement.^82^ Additionally, collaboration, adaptability, cultural competence, and an experimental mindset are essential for enhancing student learning and keeping pace with educational advancements.^68^ Education is no longer solely about delivering information but about actively engaging students in the learning process (see section How We Teach). Pharmacology educators must be bold, creative, and willing to take risks, testing new teaching methods and incorporating digital tools to enhance student learning. However, this requires a growth mindset, as well as institutional support for educators to pilot and evaluate new approaches effectively. As higher education becomes increasingly internationalized, pharmacology educators must develop cultural competence to effectively teach students from diverse backgrounds. Differences in cultural attitudes toward hierarchy, communication, and learning styles can shape classroom dynamics and student engagement.^59^^,^^83^ Educators must therefore adopt inclusive teaching practices, ensuring that pharmacology education remains accessible and relevant to a global student body.

Collaboration plays a fundamental role in advancing pharmacology education, allowing educators to develop, refine, and enhance their teaching methods while reducing the pressures of content creation. These collaborations can occur at multiple levels: within departments, across faculties, nationally, and internationally. Collaboration with educational experts can support pharmacology teachers by offering sophisticated teaching strategies, novel instructional methods, and specialized assessment techniques designed to improve learning outcomes. These experts can provide valuable guidance on curriculum design, student engagement strategies, and the effective use of technology in the classroom. Moreover, collaboration with educational specialists can promote professional growth, helping teachers to stay updated with the latest educational trends and continually refine their teaching skills.

At the institutional level, new educators often begin by forming links with colleagues teaching related subjects, such as physiology, microbiology, chemistry, and clinical medicine, enabling a constructivist approach that scaffolds learning and prevents unnecessary duplication in curricula.^26^ While in healthcare education, interdisciplinary collaboration between basic (preclinical) and clinical pharmacology educators helps vertically integrate content and maintain continuity of pharmacology knowledge across training stages.^26^^,^^84^ Studies have shown that educators who are collaborating to design and implement integrated curricula feel more supported when they are housed in interdisciplinary educational departments than they do as singular educators in their own scientific departments.^85^ In these environments, their contributions to the institution are more easily recognized by their peers, and promotion pathways are usually clearer. However, in integrated departments it can be difficult to collaborate with other pharmacology educators because they are usually limited in number. Therefore, technological advances such as web conferencing and asynchronous teaching enable pharmacology educators to collaborate with colleagues from other institutions and countries for both educational research and teaching. These collaborative networks are called virtual communities of practice.

In addition to virtual communities of practice, more formal organizations are growing at the national and international levels to support pharmacology educators. For example, the International Union of Basic and Clinical Pharmacology (IUPHAR) hosts an Education Section (IUPHAR-Ed), and many national pharmacology societies^86^ have distinct education divisions (see Table 3). Such organizations organize conferences and offer online proceedings where pharmacology educators can network to share educational best practices and collaborate in educational scholarship endeavors.

**Table 3:** Examples of pharmacological societies with dedicated pharmacology education committee

Society	Education Section/Division/Committee
IUPHAR—oversees over 60 member societies and over 100,000 pharmacologists worldwide	https://iuphar.org/pages/educations
ASPET - The American Society for Pharmacology and Experimental Therapeutics	https://www.aspet.org/aspet/membership-community/divisions/division-for-pharmacology-education-%28dpe%29
ASCEPT - Australasian Society of Clinical and Experimental Pharmacologists and Toxicologists	https://www.ascept.org/special-interest-groups/
SBFTE - Brazilian Society of Pharmacology and Experimental Therapeutics	https://sbfte.org.br/iniciativas-educacionais/
BPS - British Pharmacological Society	https://www.bps.ac.uk/about-the-bps/our-people/committees-groups/
CSPT - Canadian Society of Pharmacology and Therapeutics	https://pharmacologycanada.org/education-committee
CNPHARS - Chinese Pharmacological Society	https://www.cpa.org.cn/?do=enclass&classid=366
NVF - Dutch Pharmacological Society	https://www.nvfarmacologie.nl/education/
EACPT -The European Association of Clinical Pharmacology & Therapeutics	https://eacpt.org/working-groups/education/education-sub-committee/
PSJ - Japanese Pharmacological Society	https://pharmacol.or.jp/educator

## III Evidence-based approaches to determining what we teach and what students need to learn

What we teach and what students need to learn is described in curricula. A curriculum is a prescriptive content that illustrates what will be taught in a given educational program (input or subject matter), who will teach (teacher), who will be taught (learner), with what tools and in what context (milieu), with what effect (output/outcomes), and how that will be assessed (assessment).^87^ Although later sections will focus on the mode of teaching and assessment, this section will focus on curriculum design in terms of content selection, alignment with necessary standards and curriculum refinement.

### A Professional/accreditation requirements

Program curricula are generally determined by national requirements and where applicable, accrediting bodies. National organizations usually determine the standards required for different qualifications (diploma, degree, etc), for example, the Australian Qualifications Framework^88^ in Australia, The National Framework of Qualifications Ireland,^89^ and the Quality Assurance Agency (QAA) for Higher Education^90^ in the United Kingdom. Many frameworks (49 countries) are guided by the Bologna Process.^91^ For health professions such as nursing, medicine, pharmacy, dentistry, and veterinary medicine, curriculum requirements are largely determined by professional/accrediting bodies such as the Liaison Committee for Medical Education, which is responsible for the accreditation of allopathic medical schools in the United States and the General Medical Council (GMC) accrediting UK medical degree programs. Many of these bodies have developed competency frameworks and graduate outcomes statements that stipulate what students must be able to do/show to apply for registration. For example, in the United Kingdom the GMC provides a list of outcomes for graduates^92^ and the Adult-Gerontology Clinical Nurse Specialist Competencies include several pharmacology specific competencies that must be demonstrated before nurses can be certified as clinical nurse specialists.^93^

Within Bachelor of Science (BSc) programs, pharmacology can be included as either a major or a minor element. BSc degrees are generally not accredited by professional bodies, although they do need to comply with national standards for science programs and qualifications in general. For countries participating in the European Higher Education Area, the Bologna process provides a qualifications framework that stipulates that a Bachelor’s degree should include 180–240 European Credit Transfer and Accumulation System credits, be approximately 3–4 years in duration, and meet the following generic learning outcomes:

• “have demonstrated knowledge and understanding in a field of study that builds upon their general secondary education, and is typically at a level that, while supported by advanced textbooks, includes some aspects that will be informed by knowledge of the forefront of their field of study;
• can apply their knowledge and understanding in a manner that indicates a professional approach to their work or vocation, and have competences typically demonstrated through devising and sustaining arguments and solving problems within their field of study;
• have the ability to gather and interpret relevant data (usually within their field of study) to inform judgments that include reflection on relevant social, scientific or ethical issues;
• can communicate information, ideas, problems, and solutions to both specialist and nonspecialist audiences;
• have developed those learning skills that are necessary for them to continue to undertake further study with a high degree of autonomy.”^94^

National level guidance is provided in some countries. For example, the UK’s benchmark statement on Biomedical Science and Biomedical Sciences (QAA, 2023) provides guidance on what should be included in BSc degrees. A recent update of these QAA benchmark statements highlighted that BSc pharmacology undergraduates should possess the core knowledge, understanding, and skills associated with many of the 24 core concepts recently published by global pharmacology educators.^69^^,^^95^

### B Curriculum development

Although guidelines provided by governments and accrediting bodies, provide direction, they are often written in broad terms, requiring pharmacology educators to make important decisions about what specific content and skills to include through curriculum development. Curriculum development is a process of analysis, design, implementation, and evaluation. There are many models used to develop curricula for medical and health sciences programs, for example, the Kern 6 step model, the systemic curriculum and instructional development model, the curriculum centered strategic planning model.^96^ However, at their core, they generally include needs assessment, teaching and learning strategies, assessment processes, and evaluation processes.^96^ Although teaching and learning strategies and assessment are covered in section entitled How We Teach, here we will focus on needs assessment and evaluative processes.

#### 1 Identifying the learning outcomes required in our programs

Needs assessment informs the formulation of expected learning outcomes. Learning outcomes are statements of what students are expected to know after successfully completing a course/program. For some professional courses, learning outcomes are defined at organizational, regional, national, or international levels.^97^^,^^98^ In some courses, there may be more flexibility in designing the curriculum but guidance on required competencies and learning outcomes is often available from learned societies such as the European Association for Clinical Pharmacology and Therapeutics (EACPT), BPS, the American Society for Pharmacology and Experimental Therapeutics (ASPET), and Australasian Society of Clinical and Experimental Pharmacologists and Toxicologists (see Table 4).^69^^,^^95^^,^^97^^,^^99^, ^100^, ^101^, ^102^, ^103^, ^104^, ^105^, ^106^, ^107^, ^108^, ^109^, ^110^, ^111^, ^112^, ^113^, ^114^ In either case, learning outcomes are chosen based on experience and expertise of the education providers, competencies required for practicing a specific profession that students can transition into, transversal competencies such as problem solving and communication skills, and the rules and regulations of relevant organizations and authorities. A clear description of roles and responsibilities that graduates take up in the labor market is often a starting point^114^^,^^115^ and where this is not provided by professional bodies, this information is often gathered during the curriculum review process through interviews with potential employers.

**Table 4:** Guidance from societies and health related bodies for curriculum development

	Body/Society	Type of Guidance	References
Curriculum	BPS	Undergraduate Core Curriculum for pharmacology students	Wallace et al, 2021^99^ BPS, 2025^100^
		Clinical Pharmacology and Therapeutics curriculum for medical students (2012)	Ross and Maxwell, 2012^101^
		Curriculum for the user of research animals	Bailey and Edmead, 2023^102^ BPS, 2025^103^
		Clinical Pharmacology and Therapeutics curriculum for medical students (2026)	Lonsdale et al, 2026^104^
	ASPET	Pharmacology knowledge objectives	Theobald and Blumer, 2023^105^ ASPET, 2024^106^
	EACPT	European Core Curriculum in Clinical Pharmacology and Therapeutics for medical students	Brinkman et al, 2018^107^ EACPT Education Working Group, 2025^108^
Content guides	WHO	Model list of essential medicines	World Health Organization, 2023^109^
	IUPHAR	Core concepts of pharmacology	Core Concepts in Pharmacology, 2024^110^ Guilding et al, 2024^95^ White et al, 2023^69^
	EACPT	Key medicines list for Clinical Pharmacology and Therapeutics	Donker et al, 2024^111^
Competency frameworks	BPS	Core Skills Statements	BPS, 2023^112^
	Royal Pharmaceutical Society	Prescribing Competency Framework	RPS, 2021^113^
	NuPhaC	Competency framework for nurses on pharmaceutical care	Dijkstra et al, 2021^114^
	NPS MedicineWise	Prescribing competencies framework	MedicineWise, 2021^97^

NPS, National Prescribing Service; NuPhaC, Nurse and Pharmaceutical Care; WHO, World Health Organization.

Several societies provide further guidelines on the structure and content of pharmacology curricula (Table 4) as well as the competencies that graduates need. Although the curricular documents and content guides tend to focus specifically on pharmacology, the competency frameworks are usually more multidisciplinary but are valuable resources for pharmacology curriculum development. Most of these documents have been produced with input from educators in multiple countries. For example, Nurse and Pharmaceutical Care (NuPhaC) included experts from 14 countries when they developed a competencies framework that links specific tasks and responsibilities for nurses in pharmaceutical care to learning outcomes, including learning outcomes on pharmacology.^114^ In addition, many have used systematic approaches to reach consensus. For example, the Delphi process has been used to reach expert consensus by the BPS in the United Kingdom to create a pharmacology curriculum framework,^99^ EACPT to develop a list of learning outcomes for clinical pharmacology^107^ and IUPHAR to develop the core concepts of pharmacology (CCP).^69^^,^^95^^,^^116^

#### 2 Evaluating and refining the curriculum: Strategies for continuous improvement

Once designed, curricula need to be implemented and continuously evaluated and refined. This can be done at both a local or national level and pharmacology educators have a role to play in each. Feedback from students, staff, practitioners, external examiners, accrediting bodies, and employability liaison stakeholders ensures that programs continuously adapt the curriculum to evolving demands of employers and professional bodies. Through these efforts, pharmacology educators help ensure that future healthcare professionals and scientists are well equipped for the workforce.

At a local level, curriculum can be monitored, in the short term, through course evaluations and monitoring of assessment outcomes. Over the longer term, the extent to which learning outcomes are effectively integrated in curricula can be determined by questioning graduates after a few years of work experience about the educational program or assessing competencies in students and professionals. A summary of evaluation methods that have been used to evaluate pharmacology curricula for various purposes is captured in Table 5.^117^, ^118^, ^119^, ^120^ Such methods can help to refine and update learning outcomes.^63^^,^^121^^,^^122^

**Table 5:** Examples of evaluation strategies relevant to the review of pharmacology curricula

Program	Purpose	Evaluation Data Gathered	Reference(s)
4-year BS Pharmacy Program	Curriculum review and redevelopment	Survey of graduating student	Doria, 2017^117^
		Survey of academic staff	
		Survey of administrators (leadership)	
		Survey of alumni	
		Survey of graduate employers	
PharmD	Course review	Analysis of course reports	Aljuffali et al, 2024^118^
		Student evaluations	
		Analysis of examination center reports	
BPharm	Exploration of the curriculum for opportunities for enhancement	Content analysis of course documents using Leximancer	Noble et al, 2011^119^
Medicine	Comparing a new curriculum to the old one	Student performance on matched questions in local examinations Student performance in national examinations	Click et al, 2025^120^

For national, accrediting body or university level reviews, wider stakeholder involvement is recruited to interpret the data collected and develop new curricula. With the review of curriculum, educators must also assess whether the curriculum meets industry and workplace trends to prepare students for real-world pharmacological challenges, such as polypharmacy and emerging drug therapies. The role of pharmacology educators is instrumental to ensure that core pharmacology concepts are effectively maintained within medical, healthcare and life science programs, striking a balance between theory, practical, and clinical application. When the discipline of pharmacology is not represented strongly enough in curricula discussions, pharmacology learning goals can be underrepresented in the curricula leading to negative consequences in future years.

At a national level, pharmacology educators play an important role in advocating for pharmacology and its values in evolving curricula. Without strong advocacy for pharmacology, professional accreditation and regulation changes can, and have, drastically shaped pharmacology education in healthcare programs. In the United Kingdom, the GMCs “Tomorrows Doctors” (2002) shaped medical education for decades, outlining the essential outcomes and competencies expected of medical graduates. Although the original version of “Tomorrow’s Doctors” was published in 1993, it was the 2002 edition that had a particularly significant impact on the role of pharmacology in medical education. In this update, the document laid out a broad framework for medical education but gave limited attention to pharmacology and the teaching of drug-related topics. It largely emphasized other clinical areas, such as communication skills, ethics, and basic science, whereas pharmacology was not given the prominence it needed. Possibly as a result of this downplay, medical schools began to reduce the amount of pharmacology content within their curricula, and fewer students were being trained in pharmacology as a specific discipline. This shift led to concerns among educators and professionals that medical graduates were not being sufficiently prepared to manage the complexities of pharmacological treatment in clinical practice.^42^ The importance of having the voice of pharmacology educators represented in decisions around curricula at a national level was highlighted by a past president of the BPS when he noted that “related to *Tomorrow’s Doctors* [5], there was a reorganization of many medical schools, with loss of the undergraduate scientific disciplines, including pharmacology. Although many universities still have pharmacologists on their staff, many do not have an overarching physical or virtual organization that encompasses pharmacology and therapeutics. For clinical pharmacology to thrive, strong links with pharmacologists are essential.”^123^

So how can pharmacology educators advocate for pharmacology education? Often this advocacy happens via learned societies, such as the BPS, ASPET, Australasian Society of Clinical and Experimental Pharmacologists and Toxicologists, and EACPT. These societies are often consulted regarding national imperatives and members are given opportunities to contribute. For example, the BPS has submitted statements to the UK parliament regarding the use of animals in pharmacology teaching.^124^ ASPET provides many opportunities and resources for people who are interested in advocating for pharmacology, such as and advocacy action center with ideas (and links) on how people can advocate for pharmacology, and Capitol Hill days for all members to advocate for pharmacology in person.^125^ Advocating for pharmacology is also embedded in their strategic plan.^126^

### C Impact of evolving curricula on pharmacology education

#### 1 Evolving pharmacology knowledge means that pharmacology curricula need to evolve

Advances in drug development have resulted in an increasing number of new drugs entering the global marketplace, with over 30 new drug approvals annually in the United States.^127^ In addition, an explosion in molecular and cellular biology approaches to drug discovery and development, sophisticated target-based drug design and approaches such as phenotypic screening has led to a large number of medicines with novel structure and function.^128^ For example, mRNA vaccines proved essential tools in the rapid response to COVID-19^129^ but add to curricular load, as do novel small drug molecule mechanisms such as allosteric modulators and biased agonism.^130^ In addition, the field of pharmacology has expanded from a field focused on individual receptors and pathways, to include pharmacogenomics, computational pharmacology, systems pharmacology, and immunopharmacology, to name a few.

The inclusion (or not) of this new knowledge into curricula needs to be done in ways that ensure our graduates are equipped to work on the cutting edge of science and medicine. Such evolving curricula will require educators to engage with novel mechanisms of action and drug technologies. Traditional teaching methods may not be suitable. For example, biologics are well established therapeutic modalities and are effective in the treatment of many conditions; however, a recent review of the knowledge areas and competency standards required for pharmacists and pharmacy students concluded that “the current didactic material on biologic medicines in pharmacy degrees is not commensurate with their structural, functional, manufacturing, and clinical complexities compared to small molecule drugs”.^131^ In addition, a recent evaluation of students’ understandings of pharmacodynamic concepts noted that many students assume that a drug target is referring specifically to a protein or even a receptor.^80^ Considering the success of nonprotein drug targets as therapeutics (eg, antisense oligonucleotides and small interfering RNA), such misconceptions could impact students’ future learning about important drugs. Curricula need to evolve to ensure that learning outcomes reflect modern ways of thinking about and applying pharmacology while maintaining relevance for practice. Care needs to be taken to ensure that new content is relevant for the cohort in question to avoid curricular bloat and cognitive overload of students. Although this may seem to be a challenge, it also provides an opportunity for educators to reimagine how they teach pharmacology.

#### 2 Evolving roles of health professionals need evolving pharmacology curricula

Over the last decade, healthcare professionals’ roles in pharmacotherapy have undergone a major evolution. In particular, nonmedical prescribing, where drugs are prescribed by health professionals other than doctors or dentists, is increasing, with nurses and pharmacists now prescribing in many countries.^132^, ^133^, ^134^ In 2020, a scoping review encompassing several sources of information identified that 54% of 216 countries reviewed had nonmedical prescribing policies.^135^ Nonmedical prescribing students have stressed the importance of pharmacology knowledge in achieving prescribing confidence.^136^ During their education, healthcare professionals need to be trained to master the competencies they need in the labor market. Therefore, evolving and newly emerging roles require adaptations in the curriculum, and because of regional variations, any adaptations must be context specific. In the United Kingdom, new pharmacist training standards are currently being introduced to incorporate the skills, knowledge, and attributes for prescribing so pharmacists can independently prescribe from the point of registration putting them on a similar footing to medical graduates.^137^ The development and delivery of education and training to enable pharmacist prescribing are due to commence in 2025 in Ireland, with an aim to roll out independent pharmacist prescribing by 2027.^138^ Although education for nonmedical prescribing of pharmacists and nurses now seems to be embedded in curricula in many countries, this education still seems to require additional courses for physiotherapists, podiatrists/chiropodists, paramedics, optometrists, and radiographers. Further, although legislations and timelines have been set out for some countries, guidance on training these professionals in prescribing is difficult to find. This may indicate an opportunity for pharmacology educators to develop international overviews as a first step to prevent educators needing to start from scratch to develop curricula and courses.

Advances in pharmacogenomics and drug development have also broadened the knowledge base required by healthcare professionals. For example, in 2024 a study showed that 29% of 266 US advanced practice nurses had ordered a pharmacogenomic test within the past year. Healthcare providers are indicated to have low familiarity and a lack of knowledge in pharmacogenomic testing slowing down implementation in clinical practice.^139^^,^^140^ There have been calls for more education on the topic.^141^ Guidance on the inclusion of this topic in curricula is beginning to emerge, with the bodies from multiple countries and disciplines including pharmacogenomics in curricula and competency frameworks focused on genomic medicine.^142^, ^143^, ^144^, ^145^ Because this field is currently evolving within medical practice, it may be somewhat of a moving target, however interim learning outcomes should be defined, considering expectations regarding the roles of various health professionals in different contexts. These learning outcomes could inform the construction of course contents, the selection of teaching methods, and the creation and validation of assessments.

#### 3 Evolving laboratory practicals

In many countries, there has been a push to a more sustainable approach to laboratory practicals, including decreasing the number of animals used in teaching^146^ and adapting laboratories to be more environmentally friendly.^147^^,^^148^ This is largely due to development of a set of principles for the ethical use of animals called the 3Rs—replacement, reduction, and refinement. In many countries, implementation of these principles is included in legislation and is closely regulated.^149^ As a result, many practicals have been replaced with in vitro models and simulations.^150^ An important principle here is to ensure that the learning outcomes required by a given cohort guide the choices of whether to use a particular approach (eg, use of animal experiments). In vivo skills are still important for some pharmacology graduates to develop and there are certain concepts that are better taught with a whole organism.^151^^,^^152^ A proposed compromise is to use animals not covered under legislation,^153^ such as *Lumbriculus variagatus,*^154^^,^^155^ Zebrafish (*Danio rerio*)*,*^156^ cockroaches,^157^ and the Fruit fly (*Drosophila melanogaster*).^158^ Although the most common animals used in pharmaceutical industry are mammals, using invertebrates as an alternative allows students to observe the effects of drugs in a whole organism and learn how to work with animals while applying the principals of ethical use of animals (3Rs).

#### 4 Evolving curricula for bachelor of science programs

Although most BSc programs have no pharmacology-specific accreditation requirements, these programs need to prepare students for an evolving workforce. One exception to this is biosciences programs in the United Kingdom, which have been required to include pharmacology as a core subject and subject specialty since the release of the 2014 QAA Biomedical Sciences Benchmark Statement on Biomedical Science degrees. These benchmarks underpin accreditation of bioscience degrees by the Royal Society of Biology^159^ and BSc Biomedical Science degrees accreditation by the Institute of Pharmacy and Biomedical Science.^160^ Although this inclusion is a welcome development in acknowledging the importance of pharmacology as a discipline, BSc curricula in the United Kingdom have had to evolve to meet these new benchmarks.^99^

#### 5 Embedding sustainability in pharmacology for all programs

In addition to the need to employ more sustainable models and practices within the laboratory practicals, there is also a push to ensure that sustainable practices are embedded into degree programs with discipline-specific consideration. For example, in pharmacy, there have been longstanding efforts by International Pharmaceutical Federation to introduce students to green pharmacy practice.^161^^,^^162^ In the context of pharmaceutical sciences, green chemistry encourages critical thinking about the environmental and social impacts of drug development and healthcare. Incorporating this into curriculum provides students with opportunities to evaluate the full life cycle of pharmaceuticals; from raw material sourcing and synthesis to drug disposal and environmental persistence. Embedding green chemistry into teaching helps future professionals design safer, less toxic drugs and adopt cleaner, more resource-efficient manufacturing processes. It also promotes ethical decision-making, encouraging the development of health solutions that are both effective and environmentally responsible.

### D Content selection: Balancing core knowledge and emerging topics

The ever-evolving accreditation requirements, employer demands, and pharmacology knowledge explosion makes it difficult to select the most appropriate content. Fortunately, there are some evidence-based resources that help us narrow the content down (Table 6).^100^^,^^106^^,^^107^^,^^110^^,^^112^^,^^163^, ^164^, ^165^, ^166^ Rangachari, a doyen of pharmacology education, provides sage advice for educators here, with the concept of the nonembarrassing curriculum. This idea involves consolidating the nonnegotiable learning to “a list of items/concepts that would be so basic and general that a lack of appreciation or awareness would be an embarrassment to the program”.^167^ Luckily for new educators, international consensus on the core concepts is emerging.

**Table 6:** Pharmacology related skills in health professions and science programs

Program	Skill Type	Subtype	Example of Skills	References
Health professions	Evidence based practice	Critical reading	Safe use of national formularies (both online and hardcopy), safe use of clinical guidelines	Banning, 2003^163^ Baldwin et al, 2012^164^
		Information literacy	Identify and use information from appropriate and reliable sources	Brinkman et al, 2018^107^
		Scientific literacy	Reading medical and scientific articles	
		Critical thinking	Treatment selection, clinical (therapeutic) reasoning, rational prescribing	Brinkman et al, 2018^107^ Richir et al, 2008^165^
	Communication skills	Technical writing	Reporting adverse effects, prescription writing	Baldwin et al, 2012^164^ Brinkman et al, 2018^107^
		Patient centered writing	Care and discharge plans	Banning, 2003^163^
		Patient education	Medication teaching plan, patient education on the use and side effects of drugs	Baldwin et al, 2012^110^^,^^164^ Banning, 2003^163^
	Technical skills	Practical skills	Medication reconciliation, recording patient medication history	Brinkman et al, 2018^107^
		Quantitative skills	Dosing calculations/adjustments	Banning, 2003^163^ Brinkman et al, 2018^107^
Scientific programs	Evidence based practice	Information literacy	Identify and use information from appropriate and reliable sources	BPS, 2023^112^
		Critical thinking	Critically evaluate information from various sources	BPS, 2023^112^
		Scientific literacy	Reading	Fajt, 2008^166^
	Communication skills	Scientific writing	Integrate information from a range of sources, accurately record, and reference source material	BPS, 2023^112^
		Presentation skills	Communicate findings effectively	Fajt, 2008^166^ BPS, 2023^112^
		Technical writing	Organize and accurately record information	BPS, 2023^112^
	Technical skills	Experimental design skills	Hypothesis formulation, experimental planning and design, methodology selection	BPS, 2023^106^ Fajt, 2008^166^
		Laboratory skills	Precision and accuracy, good laboratory practice, in vitro techniques, in vivo techniques	BPS, 2023^112^ Fajt, 2008^166^
		Quantitative skills	Collect, process, and present data, apply and interpret appropriate statistical tests correctly, and use common statistical software	BPS, 2023^112^ Fajt, 2008^166^
		IT skills	Ability to access and manage computers and digital information	Fajt, 2008^166^
	Attributes	Interpersonal skills	Teamwork, working independently	BPS, 2025^100^

#### 1 The core concepts of pharmacology

In a pilot study, a group of educators from Australia and New Zealand identified 19 core concepts using a Delphi method.^168^ Subsequently, an international CCP group was established under the banner of the IUPHAR-Ed. The IUPHAR-Ed CCP built on the Australasian work to identify 25 CCP, 15 of which were present in the Australasian study.^69^ The IUPHAR-Ed CCP, then refined to 24, defined, and unpacked the core concepts.^95^ These studies used a combination of multiple rounds of Delphi method using pharmacology experts and the use of text mining of common pharmacology texts.^69^^,^^168^ Recently, over 700 students from 11 countries have completed free-text quizzes requiring the definition and application of the core concepts; analyses of the responses identified a large number of misconceptions held by students about these concepts.^79^^,^^80^ These core concepts provide insight into what students should be able to do in a nonembarrassing curriculum, leaving time for deep dives into critical concepts and novel mechanisms alike. Rote learning of intracellular mechanisms of actions for many drugs, long lists of adverse effects, and indeed essential drug lists that run into the many hundreds, are all tasks that invite surface learning approaches that do not last longer than the examination period. A focus on the CCP, as exemplified by the most important drugs as representatives of their class, is likely to leave the learner better prepared for their professional context than the “cover everything” curriculum.

#### 2 Skills in the curriculum

The depth and breadth of pharmacology is so vast, and it continues to grow. We cannot include all existing pharmacology and most certainly cannot predict what students will need to know in the future. What we can do is give students the tools and knowledge they need to be able to access, evaluate and apply the information when they need it. Although some of the skills needed by graduates are multidisciplinary, there are some that have a clear relation to pharmacology. Although the core concepts cover the knowledge students need to succeed, we also need to think about the core pharmacology related skills needed by graduates to succeed in their careers.

Despite some minor overlap between the pharmacology related skills required in curricula designed for health professionals compared with science programs, there seem to be some obvious differences. Pharmacology-related skills taught in programs focused on health professions education (Table 6) largely align with prescribing skills but could be broken down into evidence-based practice skills (such as critical reading and information literacy), communication skills (such as technical writing, patient centered writing, performing a therapeutic consultation, and patient centered education) and technical or practical skills (such as quantitative skills or medication reconciliation). Pharmacology skills needed for science programs can also be grouped into evidence-based practice skills, technical skills, and communications skills. Although the evidence-based practice skills required may seem much the same, the difference here is that the types of resources used may be different. Technical skills for science students focus mainly on experimental design skills, laboratory skills and quantitative skills relating to research activities. The communication skills highlighted in the literature for science students tend to focus on academic/research activities, however with advancements in patient-public inclusion and engagement in research, students may also need to develop more patient-facing communication skills in pharmacology.^169^^,^^170^

In professional programs in some countries there is currently a lack of education on skills relevant for prescribing.^64^^,^^171^ In most medical curricula, these skills are mainly taught by clinical pharmacologists during an integrative module in the clinical years of the medical curriculum. However, there is little exposure of skills training with simulated or real patients in clinical practice. It is perhaps not surprising that a large proportion of medical students have never written out a prescription (under supervision) for a patient during their medical training.^172^ This is concerning because students must perform these skills from the first day after graduation. In contrast, recognition of these issues in the United Kingdom led to the establishment of a Safe Prescribing Working Group (2007) under the auspices of the GMC and medical schools, which identified the need for an agreed set of learning outcomes and skills related to prescribing^173^ and a Prescribing Safety Assessment to ensure that graduates meet them.^174^^,^^175^ This has promoted a much higher focus on the importance of these aspects within undergraduate medical curricula.

Many of the skills required by medical and science graduates have not changed; however, the way they are taught has. In the past many of the skills required for applying pharmacology in a real-life context were learnt “on the job” (eg, prescription writing). However, this does not necessarily improve their knowledge and skills^176^ and in a real-life context, mistakes could lead to real consequences. Innovative teaching methods, such as case-based teaching, team-based learning (TBL), simulation-based learning, and interprofessional education^107^^,^^177^ (see section How We Teach for more detail), mean that these skills can initially be practiced in low stakes environments while students are learning. Although a shift toward skills training within the degree is essential, the resource-intensive nature of such teaching poses challenges for departments with a small number of pharmacology educators.

Skills training often poses a challenge for educators, who may not have experience of the applied skills. Although most pharmacology educators should have experience with more generic skills such as information literacy, critical reading, etc, there may be a steep learning curve when pharmacology educators need to train students in skills they have never used professionally, for example, a clinically trained pharmacology educator training students in laboratory skills. Also, recent trends toward integrated curricula and authentic assessment mean that skills are often embedded within a real-world context. Consequently, a laboratory pharmacologist who has never written a prescription in a professional capacity, may need to contribute to or mark assessment tasks or teaching activities involving prescriptions or prescription writing. One shortcoming of the studies on skills training needed within the curriculum or skills that pharmacology graduates should have, is that they are focused on a local level. With more and more globalization, it is important for qualifications to be acceptable internationally and so international consensus on which pharmacology-related skills are important would be of benefit.

## IV How we teach

### A Research-informed education—constructivism and active learning

Pharmacology has been taught in myriad ways, most commonly through didactic (lecturer-centered) lectures but increasingly through a range of more student-centered approaches. Over the past 100 years, an enormous body of research has shed light on the ways that students learn, and consequently the approaches to teaching and assessment that are most effective. These insights are worth exploring briefly for any new educator before considering approaches specific to their pharmacology context. Cognitive psychologist Vygotsky^178^ demonstrated that children learn new concepts through self-discovery, and that past and current experiences and social environments shape learning more than novice educators might expect. Our best understanding of effective pedagogy posits “an approach to teaching and learning based on the premise that cognition (learning) is the result of ‘mental construction.’ In other words, students learn by fitting new information together with what they already know.”^179^ In biological sciences such as pharmacology and physiology, students must use complex concepts to solve multifaceted clinical and scientific problems. Research suggests that when getting students to “build, test and refine” their conceptual models in a new area, the paradigm of transferring knowledge from expert to student, using lectures, is far inferior to an active learning approach in which students work together to find solutions.^180^^,^^181^ Physics educators discovered empirically that graduates of highly regarded universities could not solve problems requiring understanding of physics concepts, and indeed displayed misconceptions similar to young children, after didactic instruction.^182^ The findings of Arons and Holbrow,^183^^(p. 305)^ illustrated in this quote, support this idea:

I point to the following unwelcome truth: much as we might dislike the implications, research is showing that didactic exposition of abstract ideas and lines of reasoning (however engaging and lucid we might try to make them) to passive listeners yields pathetically thin results in learning and understanding – except in the very small percentage of students who are specially gifted in the field.

These findings led to a transformation of teaching in that discipline^184^; leaders such as Carl Wieman^185^^,^^186^ developed active learning approaches, in which lectures were transformed into classes built around tasks that required students to use the most important concepts to solve problems and predict outcomes.^183^ Mazur and colleagues, recognizing the power of peer instruction and teamwork, developed active learning approaches that centered around students working with each other.^187^ Despite progress in active learning strategies, recent evidence suggests that some educators in physics still rely heavily on didactic (teacher centered, lecture based) approaches as part of their teaching.^188^

Biology educators followed suit, developing a “Vision and Change” approach, identifying core concepts and building resources and curricula to support educators and students.^189^^,^^190^ As the use of active learning in higher education increased, so did the evaluation of these approaches. In a meta-analysis of 225 studies comparing student performance in undergraduate science, technology, engineering and medicine (STEM) courses under traditional lecturing versus active learning, Freeman et al^191^ reported that active learning improved examination performance by an average of 0.47 SDs (*n* = 158 studies) and reduced failure rates, with students in lecture-based classes being 1.5 times more likely to fail (odds ratio, 1.95).

So, what of pharmacology? Content delivery within pharmacology education has in the past relied on traditional teaching methods. Recently, there has been a profound shift toward student-centered teaching approaches, but approximately 39% of European medical schools exclusively use traditional teaching methods.^64^ This emphasis on traditional methods in pharmacology education has also been observed in the United States.^171^ Many concepts and pharmacology related competencies (eg, rational prescribing) are complex and demand advanced cognitive abilities,^192^ and simple knowledge retention by attending lectures may not adequately prepare students for their future careers. Indeed, several studies showed that students educated primarily through traditional curricula demonstrated significantly lower prescribing competencies compared with those taught using a PBL approach.^172^^,^^193^^,^^194^ More elaborate active learning approaches combine multiple instructional approaches in a coordinated and sequential manner. For example, asking students to build a concept map of blood pressure regulation centered around arterial pressure, cardiac output and total peripheral resistance, and then using those maps to solve a series of clinical problems regarding hypertension pharmacology, is more effective than simply explaining those concepts.^195^

While transitioning to more student-centered teaching is essential, the resource-intensive nature of active learning approaches may pose a challenge for some medical schools with a limited number of pharmacology educators. Ensuring buy-in from busy educators can be challenging but can be achieved with a programmatic approach^196^ and new educators may also feel overwhelmed by the need to innovate across all their teaching. Indeed, workload concerns are a limiting factor in implementing new approaches.^197^ Innovation takes time and effort, for example, one study reported an estimate of a 33% increase in workload when shifting to a new teaching approach across an entire faculty.^196^ Approaches to mitigate this challenge include staging the innovation over multiple teaching periods, allowing for evaluation and reflection cycles^198^ and the use of peers and communities of practice^199^ to provide support, expertise and encouragement. Although near-peer education has also been shown to be beneficial and can help reduce the workload of the typically small group of educators, very few medical schools actively involve pharmacists, junior doctors and medical or pharmacy students in teaching and education.^64^

Over the past 20 years, active learning approaches have been widely (although not universally) adopted in the pharmacology context.^200^, ^201^, ^202^, ^203^, ^204^, ^205^, ^206^ In practice, active learning can take a wide variety of forms (Table 7)^195^^,^^200^, ^201^, ^202^, ^203^^,^^205^, ^206^, ^207^, ^208^, ^209^, ^210^ and considering its student-centered focus, there is a considerable overlap with types of assessment that provide student feedback (assessment for learning; see section Assessment) and those that get students involved in assessment (assessment as learning; see section Assessment). Many modern approaches to teaching pharmacology are based on constructivist principles, including case-based learning, TBL, PBL, flipped classroom, inquiry-based labs, simulation, and role play.^26^^,^^211^ As noted by Rangachari,^212^ “All the active learning strategies that are discussed in the burgeoning educational literature are really variations.” A recent meta-analysis looked at outcomes of such teaching strategies across 150 studies involving 21,000 pharmacology students.^213^ The findings suggest that a range of approaches is required to achieve enhanced student performance and experience. PBL combined with case-based learning was most likely to improve students’ theoretical and subjective test scores, TBL was most likely to improve the experimental test scores and the satisfaction score, whereas flipped classroom was most likely to improve the proportion of satisfied students. The following sections examine some of the more commonly used student-centered approaches in more detail.

**Table 7:** Selected examples of student-centered approaches to pharmacology teaching

Cohort	Innovation	Outcomes	Author
Medicine	Case-based therapeutic problem/incorrect prescription/drug identification problems.	Student perceptions of understanding, arousal of intellectual curiosity and benefit of peer learning higher in active learning group. Examination scores higher in control group.	Tripathi et al, 2015^201^
	Pharmacology concepts were incorporated into large classroom team-based active learning sessions TBLs) in cardiopulmonary and neurological systems second year modules.	Mean performance on assessment items covered in TBL was above the mean for topics taught traditionally.	Gorman, 2017^205^
	Students used the online platform PeerWise to peer create, answer, and discuss multiple-choice questions related to clinical pharmacology and clinical science course material.	Medical students who used PeerWise reported learning benefits and showed improved examination performance. Specifically, answering peer-authored questions was significantly associated with higher examination scores, even after controlling for previous academic ability.	Guilding et al, 2021^207^
Pharmacy	Students developed concept maps then use maps to solve a series of clinical problems.	Students who both prepared for and attended classes performed significantly better on examination questions that required analysis of novel scenarios compared with students who did not prepare and missed classes.	White et al, 2017^195^
	Sessions in which students applied course concepts to cases, problems, and situations.	Students’ average course grade increased 2.5%. Average ratings of the course and instructor on student evaluations each increased significantly.	Kennedy, 2019^203^
	Kahoot web-based interactive games, crossword puzzles, an instructional video, a music video, and fill-in-the-blank tables.	Students perceived Kahoot web-based interactive gaming as most valuable fill-in-the-blank activities and videos were more effective as measured by examination performance.	Sumanasekera et al, 2020^200^
Pharmacy/biomedical science	Collaborative, active learning using peer instructions.	The percentage of students achieving 50% or more of maximum points per examination question was significantly increased in questions relating to peer instructions lectures.	Carstensen et al, 2020^202^
Biomedical students	Using medical television dramas as case studies for teaching clinical pharmacology.	The authors identified that this approach encouraged student discussion of complex aspects of clinical drug use and promoted interest in pharmacology.	Baños et al, 2019^208^ Baños et al, 2024^209^
Nursing	Interactive learning materials that integrated digital technology and the active learning mechanism using interactive teaching material production software.	Active learning group’s quiz scores improved significantly more than those of the control group.	Yiin and Chern, 2023^206^
Multiple cohorts	Meta-analysis of 37 studies of the use of PBL in pharmacology courses.	The use of PBL was associated with a significant improvement of examination scores.	Liu et al, 2019^210^

#### 1 Team-based learning

TBL is a collaborative education approach that emphasizes student engagement, accountability, and the application of knowledge. It aims to transform passive learning environments into active, student-centered ones by leveraging small-group work.^214^ TBL has become widely used in disciplines where problem-solving and practical applications are essential, such as medicine, pharmacy, and other health sciences, and is recognized as an effective teaching method in pharmacology education.^26^

There are typically 3 stages to TBL.^215^ Firstly, students are responsible for completing prework such as reviewing reading materials, videos, or lecture notes. This ensures they come to class with a foundational understanding of the topic. After this, students undertake a “Readiness Assurance Process,” which assesses understanding, often through an individual readiness test. Each student takes this quiz to assess their knowledge and identify any initial misunderstandings. Then students work as a team to answer the same set of questions in a team readiness test. This teamwork builds understanding as students discuss different perspectives, defend or refine their initial answers, and arrive at a consensus. Instructors then provide feedback, clarifying challenging concepts or addressing any common misunderstandings identified during the test. This guidance ensures that all students have a solid grasp of foundational knowledge before progressing to the next phase. The third stage of TBL is the application exercises. Here, students collaborate on complex, real-world problems that require them to apply their foundational knowledge. Teams engage in analyzing, debating, and justifying their responses to questions, often crafted with several plausible answers to stimulate critical thinking. Each team then presents its solution to the class, defending their choice and learning from the reasoning and mistakes of others.

TBL has been used extensively for pharmacology education, including in medicine,^205^^,^^216^, ^217^, ^218^, ^219^, ^220^, ^221^ pharmacy,^222^ dentistry,^223^ nursing,^224^^,^^225^ physician assistant,^226^ and BSc Pharmacology.^227^ The evidence from these studies indicates positive impacts on student performance by promoting deeper engagement, better retention, and higher levels of understanding compared with traditional lecture-based learning. Zgheib et al^219^ found that medical students taught pharmacology through TBL demonstrated greater knowledge retention, particularly on challenging topics, compared with their peers in traditional lectures and that summative pharmacology results were significantly improved during the year TBL was implemented. Kim et al^217^ noted that preclinical pharmacology students scored higher on examinations and demonstrated more consistent knowledge application when TBL was used in their coursework. For pharmacy students, TBL has been used for complex topics such as drug metabolism and pharmacogenomics, where team discussions deepen understanding.^228^

TBL can foster critical thinking and clinical reasoning by engaging students in real-life clinical case exercises that connect theoretical pharmacology concepts with practical applications, reinforcing their understanding of drug actions, interactions, and side effects in a clinical context. Carrasco et al^216^ found that TBL improved decision-making skills in pharmacology, especially for students who initially struggled. By working together on these cases, students developed teamwork, communication, and problem-solving abilities, skills essential for clinical practice. El-Banna et al^224^ observed that nursing students in TBL-based courses improved these skills more effectively than those in traditional lecture-based courses. TBL’s emphasis on collaboration makes it especially suited for interprofessional education. Indeed, it has been successfully applied in pharmacology education activities, which bring together students from various healthcare disciplines.^211^^,^^229^^,^^230^

#### 2 Problem-based learning

PBL is well established as a transformative and innovative pedagogical approach that, most importantly, is student-centered. It harnesses real-world problems as a scaffold for knowledge acquisition and application.^231^ It also promotes active learning by encouraging students in small groups to collaborate, analyze and resolve complex, intricate, open-ended problems. PBL is deeply rooted in constructivist theory, which emphasizes self-directed inquiry, values collaborative discussion, and prioritizes critical thinking as essential tools that learners can use to “construct” knowledge.^231^ The methodology is predicated on 3 fundamental principles. Firstly, it employs authentic, real-world problems as learning catalysts. Secondly, it promotes small-group collaboration to nurture peer-to-peer interaction. Lastly, it integrates facilitators who guide rather than dictate the learning process.^232^ This approach stands in stark contrast to traditional didactic lectures. In PBL, students are not mere vessels to be filled with knowledge. Instead, they are the architects of their own understanding. They identify learning objectives with autonomy, conduct research with diligence, and synthesize information to develop solutions with ingenuity.^232^

Pharmacology is inherently interdisciplinary. This mandates that students have a good command of the pertinent material and be able to connect drug mechanisms with physiological processes, disease states, and therapeutic outcomes. Traditional lecture-based teaching often compartmentalizes this knowledge, making it difficult for students to see the bigger picture. PBL, however, encourages students to explore pharmacological concepts in the context of real-world problems, enabling them to make meaningful connections between theory and practice. Pharmacology classrooms especially benefit from PBL because of the complex nature of course content, which requires integration of pathophysiology, drug mechanism, and therapeutic treatment choices. For instance, in a PBL scenario, students might be presented with a case study of a patient with hypertension who is not responding adequately to their current medication. Through guided inquiry, students would explore the underlying pathophysiology of hypertension, the mechanisms of action of various antihypertensive drugs, and factors that might contribute to treatment resistance, such as drug interactions or patient nonadherence. This approach not only reinforces pharmacological knowledge but also helps students understand how this knowledge is applied in clinical practice. In marked contrast to traditional approaches, PBL encourages students to take ownership of their learning by working through realistic, case-based scenarios; this not only enhances motivation, but it also advances comprehension.^233^, ^234^, ^235^ Indeed, this active engagement has been shown to improve long-term retention of pharmacological concepts.^236^ For instance, it is documented that medical students exposed to PBL exhibited superior recall of pharmacological principles 6 months after instruction, suggesting that active engagement with material enhances memory consolidation.^237^ “One important component of PBL-based curricula is the triple jump assessment. This is a formative and summative assessment approach that offers a dynamic approach to evaluating reasoning skills, particularly clinically-related ones.”^238^^,^^239^

Healthcare is a collaborative endeavor. It requires effective communication and teamwork among professionals. In this context, one of the most significant added values of PBL is its capacity to nurture and promote collaboration skills among students. PBL fosters these competencies by requiring students to work in small problem-solving groups.^231^ For example, in a typical PBL session, learners cooperatively analyze cases, share insights, and devise solutions. Through group discussions, they hone their articulation skills, listen attentively to peers, and respectfully negotiate differing perspectives.^232^ Importantly, this collaborative environment mirrors clinical practice, whose milieu is one that involves perhaps the most interdisciplinary and collaborative teamwork, largely because effective communication and cooperation are essential for patient care.^240^ Indeed, students participating in PBL report higher levels of satisfaction with their group interactions.^241^ They also perceive themselves as better prepared for future professional collaborations.^241^ These experiences clearly demonstrate the importance of PBL activities as important avenues to better prepare future healthcare professionals for optimal patient outcomes.^242^, ^243^, ^244^

##### a Challenges and considerations in implementing problem-based learning

Despite its numerous advantages, implementing PBL in pharmacology education is not without many challenges. These include resource constraints such as limited faculty time, insufficient training, and the effort required to develop high-quality, clinically relevant cases.^245^ This can be particularly difficult in settings where many faculty members are basic scientists with little or no clinical experience. One potential strategy to address these limitations is near-peer teaching, where senior students facilitate small-group case-based or problem-based sessions. Systematic reviews show that, with appropriate training and oversight, near-peer facilitation achieves learning outcomes comparable to faculty-led teaching and offers additional benefits such as improved self-efficacy for both junior and senior students.^246^ In medical education specifically, meta-analytic evidence demonstrates that peer-assisted learning significantly enhances academic performance, particularly for clinical and practical skills.^247^ Although pharmacology-specific evidence is limited, emerging studies suggest that peer tutoring can increase engagement and confidence, and pharmacist-led peer teaching has been shown to improve prescribing skills and learner confidence, leading to national adoption in the United Kingdom.^248^^,^^249^

The time-intensive nature of PBL sessions can strain institutional schedules, particularly in curricula already burdened by extensive content requirements. Faculty members, too, face challenges in transitioning from traditional lecturing roles to facilitators of self-directed learning, a shift that necessitates specialized training and ongoing support.^242^ Additionally, PBL often requires more physical space and facilitators compared with traditional lecture-based methods, which can strain institutional resources.^26^

Another significant hurdle is student resistance to self-directed learning. Many students are accustomed to structured, teacher-led instruction. Such students, especially those in earlier years of their medical school education, may initially struggle with the autonomy and responsibility demanded by PBL.^236^^,^^250^ Some students may, therefore, feel uncomfortable with the ambiguity inherent in complex problems or struggle with the increased responsibility for their own learning. This may lead to student resistance, which can manifest as frustration, anxiety, or even disengagement.^251^ This is particularly evident among those who lack confidence in their ability to independently navigate complex problems.^240^ Additionally, too much emphasis on group work in PBL can exacerbate interpersonal conflicts or uneven participation, further complicating the learning experience.^250^^,^^252^^,^^253^

Assessment poses yet another challenge in PBL implementation. The collaborative nature of PBL and the focus on process skills (eg, critical thinking and problem-solving) rather than just content knowledge can make it difficult to evaluate individual student progress. As such, assessing student performance in PBL can be more challenging than in traditional teaching methods.^26^ For instance, unlike traditional examinations, which rely heavily on measuring recall or assessing close-ended questions, evaluating outcomes in PBL requires nuanced tools capable of capturing higher-order thinking skills, collaborative abilities, and problem-solving proficiency.^254^^,^^255^ However, designing such assessments is inherently difficult, and inconsistencies in grading rubrics can undermine the perceived fairness and reliability of evaluations.^256^ Moreover, inadequate standardized metrics for assessing PBL effectiveness makes it challenging to compare results across institutions or programs.

To address the challenges associated with PBL implementation in pharmacology education, several evidence-based strategies have been proposed. Hybrid models that combine PBL with traditional lectures offer a realistic and pragmatic solution. These models allow educators to balance the strengths of both approaches. For instance, introductory lectures can provide foundational knowledge before students engage in PBL sessions, ensuring that they possess the necessary foundational knowledge to effectively tackle complex problems.^242^^,^^257^ Moreover, evidence suggests that integrating brief didactic components with PBL significantly improves student performance on pharmacology examinations, highlighting the potential of hybrid methodologies.^244^

Another interventional mitigation or pre-emptive strategy is to run faculty development programs. Investing in training programs is crucial to equip educators to serve as skilled facilitators and assessors of PBL. Workshops focusing on group dynamics, student conflict resolution, questioning techniques, and feedback delivery can empower instructors to guide discussions without dominating them.^258^ Additionally, mentorship programs pairing experienced PBL practitioners with novices can accelerate the adoption of best practices and foster a supportive community of practice.^259^ A critical element of this training is ensuring that facilitators remain aware that their job is to facilitate, rather than dominate, the active group discussions.

To mitigate student resistance, it would be advised that PBL be introduced gradually. Moreover, providing thorough orientation for students can help address resistance to self-directed learning. This approach allows for “acclimatization” to the new learning style and develop the necessary skills for success in PBL. Providing clear rubrics that details expectations and guidelines can help students navigate the transition to a more active learning approach. Moreover, engaging students in the assessment process (eg, self-evaluation) may help them realize the importance of PBL, thus leading learners to become owners of their own educations and lifelong learning journey.

#### 3 Leveraging technology in education

The digital proficiency of today’s students has driven the adoption of technology in pharmacology education. Simulation-based learning in the form of the human patient simulator “SimMan” has been extensively used for the teaching of pharmacology and therapeutics in pharmacy, medical, and nursing education.^260^, ^261^, ^262^, ^263^, ^264^ This is often one of the first “patients” that students get to meet and treat in a “safe” space, and it has been reported to support medical student knowledge and confidence level.^261^ Other simulation-based pharmacology models, which have stood the test of time include the organ bath simulation.^265^ For decades, these models have been instrumental for teaching pharmacology students’ fundamental pharmacological principles of drug action using simulated models of guinea pig ileum, among other simulated tissue settings.^265^^,^^266^ In the field of quantitative systems pharmacology.^267^ With the advancements of predictive AI technology such as AlphaFold,^268^ it will only be a matter of time before AI use in the pharmacology education setting is realized as a way to support student learning.^269^, ^270^, ^271^ The potential of these technologies to support students with molecular pharmacology in visualizing different drug binding modes, impact of patient variation on drug targeting etc is endless.^272^ Virtual, reality technology has already proven to contribute to positive learning outcomes in pharmacology,^273^ however, cost continues to be a barrier preventing its widespread use.^274^ A more comprehensive discussion on the use of AI in education is covered in section Artificial Intelligence.

At the commencement of the COVID-19 pandemic, educators were suddenly faced with the seemingly impossible task of delivering programs wholly online. Remarkably, this was achieved at universities around the world, and pharmacology educators were at the forefront of this change.^275^, ^276^, ^277^, ^278^, ^279^, ^280^ The human toll of the pandemic should not be underestimated, with educators and students profoundly affected and mental ill-health prevalent.^276^^,^^281^ The adaptations required of educators and students dramatically increased their workload. A study at a German university found that more time was spent on study (students) or teaching-related activities (educators, estimated at an 80% increase).^276^

One of the greatest immediate challenges for institutions was to move content from classroom presentations online. A range of approaches was used, often initially involving sharing of long recorded lectures then becoming more sophisticated and including short videos integrated with quizzes and other interactive media.^282^ Another immediate challenge was to develop the technological medium by which online classes would be held. Classes transitioned to online learning in many instances. One of the greatest challenges during the pandemic was to provide students with practical experiences, placements and industry engagement.^279^

During the pandemic, simulation tools and virtual laboratory options such as Labster provided an accessible way for students to learn pharmacology in the absence of being able to access the laboratory.^283^ Although computer-based options were effective,^284^ for many pharmacology students, learning fundamental pharmacology practical skills is an essential part of their undergraduate experience.^285^ Beyond the use of simulation-based approaches, some have taken a creative approach with the use medical television dramas to teach pharmacology and engage students with key theoretical concepts.^208^ Other educators have demonstrated the value of incorporating gamification with positive impact to student engagement, retention of knowledge, and performance in pharmacology.^286^^,^^287^ The use of simulations, interactive modules, and online assessments offer dynamic learning experiences that cater to diverse learning styles.^288^ Additionally, blended learning models provide flexibility for students balancing academic commitments with other responsibilities.

Finally, assessments were transitioned to online formats at scale. Many institutions used proprietary examination software to run assessments online, invigilated remotely. In some cases, students were allowed “take home exams” that could be done in their own time. A US study found that there was no significant difference between prepandemic and pandemic examination performance among medical students in Mississippi.^7^

The American Association of Colleges of Pharmacy surveyed institutions regarding their intentions after the pandemic. Interestingly, there was a desire to continue active learning strategies and TBL for student pharmacists. Some coursework was expected to continue online, “particularly for electives, prelaboratory work and small group discussions/facilitations”.^289^

### B Assessment

Assessment refers to judgments of students work usually made in alignment with disciplinary norms. We make such judgments for 2 main reasons:

1 To provide what Knight^290^ describes as “*feedout* …in the form of grades or classifications that can be used as performance indicators for the student…” as well as for institutional quality assurance purposes.
2 To provide *feedback* that identifies gaps in student learning.

Assessment has undergone enormous change, with emphasis shifting from a focus on “feedout” or assessment *of* learning (eg, high-stakes summative assessments), to a greater emphasis on assessment *for* learning, which provides feedback (eg, continuous assessment) and assessment *as* learning (eg, self-assessment, peer assessment, etc). Although assessment of, for, and as learning are often presented as distinct types of assessment, Schellekens et al^291^ suggest that they should be considered as parts of a whole assessment approach. There has also been a shift to more authentic forms of assessment, which allow students to demonstrate knowledge, skills and attitudes that are relevant to their program of study and likely professional context.^292^ These changes have been accompanied by massive growth in the range of assessment technology available as well as increased dissemination of assessment innovations by pharmacology educators (Table 8).^175^^,^^195^^,^^292^, ^293^, ^294^, ^295^, ^296^, ^297^

**Table 8:** Examples of assessment innovations used by pharmacology educators

Cohort	Assessment Innovation	Outcomes	Author
Pharmacy	Simple cases requiring application of core concepts	Students that practiced cases and attended classes performed better on novel problems but not on recall questions	Serrano Santos, 2017^292^ White et al, 2017^195^
Nursing	Authentic scenario-based online assessments	Asynchronous elements involved peer-to-peer learning relevant to future clinical practice	Lee et al, 2025^293^
Medicine	Role play for assessing patient communication	Increased confidence in communication of medicine-related information	Lavanya et al, 2016^294^
	Assessment of prescribing competency via online patient scenarios	Validity and reliability established for national prescribing competency assessment	Maxwell et al, 2017^175^
Multiple	Simulation of pharmacology in vitro preparations with self-assessment	Free resources, increased skills in experimental design and evaluation of results	Dewhurst and Ward, 2014^295^
Health sciences	Student-developed clinical trials assessed		Rangachari, 2002^296^
	Interprofessional clinical pharmacology assessment with pharmacy residents and medical students	Peer-to-peer learning relevant to future clinical practice; gives early medical students assessment in patient specific medication problems	Schramm et al, 2017^297^

#### 1 Assessment of, for, and as learning

It is important to note that it is the context and ways in which the assessments are used that determine whether they are assessment *of, for* or *as* learning. For example, and objective structured practical examination (OSPE), can be used at the end of a module to provide “feedout” on a student’s performance, or at different points during a course to provide feedback to students that enhances learning (assessment for learning).

*Assessment of learning* refers to high-stakes summative assessments, which assess student knowledge at the end of a semester/year. In pharmacology, this can take the form of invigilated closed book examinations or objective structured clinical examinations (OSPE or OSCE). Although universities in some countries have moved to a more continuous assessment (formative)model of assessment, high-stakes summative assessments remain the dominant form of assessment in many countries.^298^ Although cost and efficiency often play a role in the inclusion of such high stakes examinations, educational reasons such as enhanced motivation to study, minimizing opportunities for cheating (eg, using AI for at home assignments) and examination validity have also been cited for the continued use of such assessments. However, a recent scoping review highlighted that although students’ motivation to study may be proportional to the % marks associated with the assessment, the type of learning encouraged is often superficial (eg, rote learning) and/or strategic (eg, only studying parts of the curriculum and the validity of many summative assessments is questionable).^298^ In addition, they highlight that high stakes assessments can negatively impact student mental health and are not the best tools for incorporating authentic assessment (see below).^298^

For curricula, where summative assessment remains a dominant feature, some practical steps can be taken. Examinations can be designed to include questions that encourage deep learning and application of knowledge and where relevant can attempt to incorporate some aspects of authenticity. However, the extent to which this can be done is limited by the examination setting.^298^ In terms of validity, educators can use; (1) Quantitative tools where relevant to determine the quality of questions^299^; (2) AI to identify ambiguous questions^300^; (3) Reflective tools to ensure that the assessment aligns with the intended learning outcomes.^301^ Although some studies in pharmacy have shown that anxiety has little or no impact on student performance in OSCEs,^302^ others have shown a negative effect on performance.^303^ Either way, the levels of anxiety experienced by students is a welfare issue. Steps to alleviate such anxiety could include the inclusion of formative or practice OSCEs.^304^ An alternative is to shift your assessment to *assessment for learning* or *assessment as learning*.

Although TBL (discussed in section Research-informed education—constructivism and active learning) provides a course or program level integration of *assessment for learning*, there are many approaches to *assessment for learning* that can be integrated at a lecture level. For educators looking to take their first steps into *assessment for learning*, audience response systems provide students with problems they can answer independently or in groups (eg, “Poll Everywhere”). These methods allow students to tackle questions that require recall, or even better, application or analysis of novel scenarios, and can be used to develop critical thinking and problem-solving skills. Although students receive feedback on their answers in class, often the most important learning facilitated by audience response systems happens during the peer discussions that precede the voting for a best answer.^305^ Another example of *assessment for learning* can be seen in student-centered teaching methods such as “Self-study, Test, Question and Discussion.” This method has been previously shown to be effective in learning enhancement in pharmacy by encouraging students to formulate questions, solve problems, and engage in discussions.^306^ Its use in an integrated basic science curriculum can improve learning skills and outcomes in pharmacy education. Self-study, Test, Question and Discussion allows for personalized teaching, requiring careful student grouping and effective management of in-class discussions. Although beneficial, one drawback is that this approach increases educator workload because of the investment of time required to produce self-learning materials and feedback. One approach that integrates assessment of, for, and as learning, called programmatic assessment is gaining ground in medical and other health curricula.^307^^,^^308^ This model focuses on collecting continuous data from different assessments over time in a more meaningful and “diagnostic” way to build a holistic picture of the learner’s competence and development. This includes a combination of low and high stakes assessments, as well as more objective and subjective measures. The focus of programmatic assessment is to provide feedback that is both regular and actionable, helping learners throughout their educational journey, rather than relying on single high-stakes examinations.^309^ The concept is based on the robust finding that competence does not generalize well across content—for example, from drug calculations to therapeutic counseling skills—but does generalize well across formats within the same content domain.^310^ This is similar to a clinician combining medical history, laboratory results and comedication with usability, drug availability, cost, and environmental impact to select the most appropriate drug treatment. Assessments typically occur at multiple points (called data points) throughout the curriculum, but do not result in individual pass/fail decisions. Instead, they are all informative, collected in a dossier or (electronic) portfolio, and periodically analyzed and discussed with a coach or mentor in a follow-up meeting.^308^

*Assessment as learning*, sometimes referred to as a subclass of assessment for learning, emphasizes the student’s active role in assessment.^311^ As well as providing opportunities for learning of disciplinary knowledge, assessment as learning, is associated with the development of skills in metacognition and self-regulation. Therefore, considering increasing emphasis globally on developing students as lifelong learners, it is not surprising that there has been an increase in its use globally.^311^ Some examples from pharmacology include, involving students in generating examination questions, for example, multiple choice questions,^207^^,^^312^ eCases,^313^ assessment criteria,^314^ self-assessment,^315^ and peer assessment.^316^

#### 2 Authentic assessment

Authentic assessment is a concept that was first described by Wiggins in the 1990s and refers to *“when we directly examine student performance on worthy intellectual tasks”*.^317^ What authentic assessment looks like will change across different disciplines, and in the case of pharmacology, vary depending on the degree program in which pharmacology is being taught. To be effective, authentic assessment should be designed to reflect real-world tasks and skills relevant to a specific field of study. Quite often authentic assessment is designed with employability skills in mind,^318^ therefore what authentic assessment looks like for a pharmacy student will not necessarily translate across to pharmacology students on a biomedical program. Although the core principle remains the same, whereby the assessment should be designed to evaluate students in a way that mirrors professional practice, the application will differ depending on the program learning outcomes. For students in life science degrees, educators are adopting the “student as partner” model for the codevelopment of authentic assessments that help to support the diversity of career pathway students take upon graduation.^319^ The participatory approach of student as partner has been previously found to empower pharmacy students by permitting the student to be involved in the design of curricula more relevant to their needs and aspirations.^320^ This has led to the rise in assessments that are situated in a simulated or even real health professions or scientific contexts.^293^

From a practical standpoint, OSCE are common practice as an authentic assessment for pharmacology students as part of medical and pharmacy programs.^208^^,^^321^ These approaches have been shown to be effective in the assessment of clinical reasoning and decision-making skills for students for over 50 years.^322^ Although not traditionally used in medicine or pharmacy, there has been an increase in the use of OSPE as a way to assess student practical competences.^323^^,^^324^ The value of OSPE assessment in evaluating application of pharmacology knowledge across nonvocational basic science cohort has recently been shown be effective in identifying student knowledge gaps.^325^

#### 3 Technology in assessment

In addition to traditional approaches such as multiple-choice questions, essays, and practical or laboratory assessments, pharmacology educators can use online quizzes with an increasing sophistication of options for automated marking and feedback,^326^ role plays, case-based or problem-based assessment,^327^ and can incorporate peer feedback either electronically or in person.^328^ Assessments now need to cover skills such as effective patient communication^294^ and more sophisticated techniques such as motivational interviewing.^329^ In basic sciences, simulation has been used extensively to assess pharmacology knowledge and skills to replace animal use.^330^^,^^331^ Although it is appealing to try these new technologies and teaching approaches, we must be cognoscente of the principles of good curriculum and assessment design. Most notably, the concept of *constructive alignment*, in which there is alignment between teaching goals, teaching methods, and assessment, allows students to make sense of the educator intention for their learning.^332^ Any assessment decisions should ensure that such alignment is maintained or even enhanced. In addition, different technologies have different affordances (what they will and will not let users do). It is important to ensure alignment between these affordances and what you want to achieve in technology enhanced assessment.

Considering the rise of generative AI, many educators are rethinking their approach to authentic assessment to preserve academic integrity.^333^ In the spirit of embracing the potential of AI as an effective learning tool, educators within computing science have designed diverging assessments that permits generative AI tool use for the purposes of enhancing student’s metacognitive knowledge.^334^ Divergent assessment in pharmacology could encourage students to explore multiple possible answers or solutions, introducing creativity, critical thinking, and application of knowledge.^335^ If effectively designed, this would allow students to consider various drug interactions, mechanisms, and treatment options, promoting real-world problem-solving and decision-making skills. By engaging in open-ended tasks such as case studies or debates, the use of divergent assessments in pharmacology could create a learning environment where students develop a deeper understanding of pharmacological concepts and improve their ability to handle complex situations, preparing them better for professional practice.^170^^,^^336^

#### 4 Assessing prescribing knowledge and skills

Prescribing knowledge and skills assessment can be incorporated in relevant degrees either as part of student learning through activities such as case-based discussions or workplace-based assessments including preprescribing, or a variety of different question types can be incorporated in summative assessments.^337^^,^^338^ Of major interest in the assessment of prescribing knowledge and skills, are the international prescribing safety assessments. Several examples of such high stakes summative assessments are now deployed such as the UK Prescribing Safety Assessment.^174^^,^^175^^,^^339^, ^340^, ^341^ The Dutch National Prescribing assessment has also been delivered,^193^^,^^342^ as have other prescribing assessments including the European Prescribing Exam.^343^ A Prescribing Skills Assessment that adopts the same blueprint as the UK model is also delivered to Australia, Canada, Ireland, Malta, and New Zealand.^344^^,^^345^ These assessments are generally created through national collaborations that bring together physicians, pharmacists, and other educators who recognize the importance of delivering a high-quality assessment and establishing appropriate standards in this complex and challenging area of practice. Undertaking such assessments on a national basis also enables groups to deliver assessments to a standard that is rarely possible in single institutions given the limited available expertise and resource.

Issues that have to be considered in planning prescribing assessments include:

1 *Coverage.* This will normally be informed by the curriculum of learning it is designed to assess but should also be rooted in the workplace that the learner is being prepared to work in (if it is known).
2 *Validity.* Is the test merely a surrogate marker of likely performance in the real-world setting (eg, multiple choice questions), a high-fidelity simulation or set in the real world.
3 *Delivery.* Remote online through to real world physical environment. Electronic or paper.
4 *Purpose.* Summative high-stakes assessments or formative exercises providing feedback, learning, and reflection.
5 *Reliability.* How confident can we be about reproducibility—this is determined by its length, item discrimination, and number of skill domains.
6 *Marking.* This should ideally be objective and consistent candidate to candidates.
7 *Governance.* Any high-stakes assessment requires a clear governance structure that oversees quality assurance (eg, assessment board), academic credibility, standard setting, and appeals processes.

The presence of these kinds of prescribing assessments ensures that there is a focus within the learning program on delivering the necessary learning outcomes, which can become lost in increasingly crowded undergraduate curricula. They also ensure that weaknesses in this vital aspect of knowledge and skills cannot be compensated for by good performances in other parts of the curriculum. A Swedish study showed that 90% of third-year medical students in one medical school passed the internal medicine examination when clinical pharmacology questions were integrated; however, only 73% passed when these questions were assessed separately.^346^ However, it is important to recognize that dedicated assessments are not universally welcomed especially by those that argue that it is inappropriate to create “special status” for some areas of the curriculum and not others. This is clearly a philosophical issue, but clinical pharmacologists might argue that the demonstrable evidence of adverse prescribing outcomes (to the detriment of patients) and deficits in training pathways, highlighted by both students and clinical supervisors, do give the assessment of prescribing skills a special status.

Such standardized prescribing safety examinations usually provide “feedout” rather than feedback to students but serve an important purpose in certifying students ability to safely prescribe and supervise the use of medicines. These standardized assessments also make it possible for educational institutions (eg, medical schools) to reflect on their training programs, which often vary considerably.^64^ If comparative data are available, educational institutions can benchmark the success of their approach against others. If specific item-related data are available from assessments, they can consider specific areas of weakness or misunderstanding and amend teaching accordingly. Prescribing is a complex skill that involves communication between the prescriber and patient, but also communication between prescriber and those who dispense the medicine (usually a pharmacist) and those who administer the medicine (usually a nurse). Development of such communication skills is increasingly a focus of teaching activities and related assessments, and increasingly sophisticated simulations have been developed for this purpose.^347^^,^^348^

### C Artificial intelligence

The idea of consciousness and intelligence being bestowed upon artificial beings by master craftsmen has existed in stories and myths since antiquity. In 1950, Turing^349^ described “learning machines” and proposed the “imitation game” as test of a machines ability to mimic human intelligent behavior through natural language conversations assessed by a human evaluator. The development of large language models (LLMs) in 2018 enhanced natural language processing capabilities leading to the launch of the conversational chatbot, ChatGPT, in November 2022.^350^^,^^351^

LLMs are sophisticated artificial neural networks, a subset of machine learning, the development of which has been inspired by the structure and function of the human brain. Given a user’s text prompt, ChatGPT and other LLMs generate a text response by predicting the next word, or token, given the context of the prompt and based on the patterns in language that it has “learned” during the training process.^352^ LLMs therefore do not reproduce text from training sources but rather generate new text word-by-word through a highly sophisticated statistical algorithm. The text generated is therefore unique, even when the same prompt is used, and constructed with good quality language.

The capabilities of ChatGPT have sparked a great deal of concern among HEIs, regulators, and the media that these technologies would negatively impact the integrity of assessment and academic standards.^352^^,^^353^ As AI technologies continue to develop rapidly there is also a growing acceptance that they have the potential to enhance learning, teaching and assessment. Many educators, however, lack the necessary understanding of how AI tools work and the implications this may have on potential applications in the classroom. Educators and students may also not be fully aware of the ethical implications and limitations when using AI tools. This AI literacy gap must be addressed to ensure teaching, learning, and assessment are conducted effectively. As educators, we also have a responsibility to develop our learners’ knowledge and skills in the responsible, critical and effective use of AI tools to prepare them to succeed in an ever more AI-enabled world.^354^ To understand the potential challenges and benefits that the AI revolution brings to higher education we need to understand how these tools work, their capabilities and limitations.

#### 1 Concerns about, and limitations of, generative artificial intelligence

Generative AI tools can already produce impressive content whether that be code, text, images, audio, or video, and with ongoing rapid development the quality of output will continue to improve. Generative AI technologies are however not perfect and have several limitations in their capabilities as well as concerns around their development and potential misuse. They are susceptible to generating outputs that perpetuate bias and false information.^355^

Although text generated by LLMs reads well and appears convincing it can contain facts that are made up and reasoning that is illogical. These are often referred to as “hallucinations” and is in part a consequence of the way in which LLMs generate text. If you were to prompt ChatGPT to include citations and a list of references within its output, it can and will do so, but these may not be accurate or indeed even exist within the literature. In the medical context, use without understanding the limitations of generative AI and critical review of their output has the potential to adversely impact patient care.^356^

Apart from the accuracy of outputs, there are other concerns associated with the development and deployment of generative AI tools. In many cases data entered as prompts into LLMs are used to train and refine the model.^357^ In addition to copyright material, the enormous data sets used to train LLMs include explicit and disturbing content found in the deepest recesses of the internet. To protect users from abusive and harmful outputs, AI tools have been trained to detect such content and prevent the likes of ChatGPT from producing it.

There is also a growing awareness of the environmental impact that large-scale generative AI models have.^358^ This includes the impact of mining for minerals and increased energy consumption to deliver the computational power required to train and deliver functionality to users. It has been estimated that current use of one platform, Stable Diffusion, could generate as much as 360 tons of CO_2_ per year.^359^

#### 2 The challenge of generative artificial intelligence and assessment

One of the most pressing challenges posed by generative AI is having confidence in the authorship of assessed work. Academic misconduct, be that collusion with a peer, submission of someone else’s work, or using essay mills, is unfortunately not a new concept. However, generative AI exacerbates the threat to integrity of assessment and academic standards by making it easier to generate new content from simple text prompts. The performance of ChatGPT in Medical Licensing and Bar examinations is well documented and highlights the capability of AI tools in interpreting and answering questions in high level assessments.^360^^,^^361^

AI tools continue to evolve and become more sophisticated and are now able to analyze images and text, integrate information from multiple sources, demonstrate critical analysis and provide reasoning for answers provided.^362^ These are of course some of the higher order skills we may seek to assess over and above factual recall. In addition, distinguishing between human and AI-generated content is increasingly challenging. The reliability and fairness of noninvigilated written assessments are therefore vulnerable to the capable of AI tools in generating increasingly accurate well written text of as good, if not better, quality than the average human. Indeed, a study in found that 97% of wholly AI generated short answer and essay-based assessments went undetected by markers and were awarded grades higher than the average student in the cohort.^363^

#### 3 Can we rely on artificial intelligence–text detectors?

There has been a rapid rise in tools that claim to detect AI generated text and provide reassurance to those concerned about the authenticity of content. Can AI-generated text be detected reliably? The short answer is no, and this is likely to be the case in the near future.^364^ In January 2023 OpenAI, the creator of ChatGPT, launched an AI classifier that was trained to distinguish between human and AI-generated text from a variety of LLMs. Within 6 months the tool was withdrawn as it was “not fully reliable” being able to correctly identify only 26% of AI-written text and falsely identifying 9% of human-written text as being AI-written.^365^ With the concerns around misuse of generative AI in assessment, it was perhaps not surprising that Turnitin developed and AI detection tool against GPT3 and ChatGPT. At launch, Turnitin claimed the tool had a successful detection rate of 97% and a false positive rate of 1 in 100. After real world application, Turnitin themselves admitted the results were different from their testing and that in some cases false positive rates may be up to 4 times higher.^366^

Although the prospect of a robust and reliable AI detection tool may provide a degree of comfort, the reality is that this is unlikely to be the case given the limitations of AI detectors.^367^ The problem with AI detectors is not limited to their real-world performance and reliability. With the evolution of existing, and development of new and more advanced LLMs, we are at risk of getting caught up in a technological arms race between generative AI and checkers… and paying for it. AI detectors work best on unaltered generative AI outputs. Paraphrasing and editing of the text, either by a human or via a paraphrasing tool, disrupts the algorithms on which AI detectors rely.^367^ There is also evidence that AI detectors are biased against nonnative English writers, with higher false positive rates compared with native English writers.^368^ With a false positive rate that is too high detection can lead to too many false accusations of cheating, causing unacceptable student dissatisfaction and reputational damage.

With generative AI technologies being embedded within many tools used in education, for example, Grammarly and Microsoft Office, it is possible that we all at some point will be producing work that includes AI generated, or AI improved content. The real question is whether we consider it feasible to fight the use of AI technologies or accept their role as an assistant and focus on the value added that we as humans can bring. Focusing on a person’s ability to use AI tools effectively and critically through expertise in prompting and enhancing outputs may become a more appropriate skill to develop and ultimately for us as educators to evaluate through assessment.

#### 4 Advice to educators

With a strong foundation in AI literacy, students will be more likely to use AI tools ethically, and it offers the opportunity to promote academic integrity and learners’ awareness of how to use AI tools responsibly and ethically. It is also necessary to review assessment practices ensuring a variety in modes of assessment and replacing highly vulnerable assessments with authentic assessment of knowledge and skills and, where appropriate, the use of closed book invigilated assessment of knowledge and skills. For example, it may be appropriate to adopt invigilated assessment in the early years of a course when assessing fundamentals, for example, basic mathematics without use of a calculator. In later years of the course, it may be more appropriate to shift to modes of assessment that allow the use of AI technologies as an assistant and focusing on the students’ ability to produce work using these tools effectively, reflecting the future workplace, for example, allowing the use of a calculator in an assessment of mathematics. AI can be used to generate clinical vignette prompts modeling adverse reactions, drug-interactions, and patient specific dosing.

#### 5 Potential roles for artificial intelligence in learning, teaching, and assessment

The use of AI tools such as MS Copilot, Gemini, and others may realize efficiency gains for educators through supporting instructional design and the development of course materials including simulations, clinical vignettes, and assessments. Successful adoption of AI as an assistant will therefore enable the educator to devote more time to more impactful learner interactions and mentoring, improving the student learning experience.

AI tools may have a role in planning a lesson structure and in the creation of associate teaching resources, doing so quickly with mostly appropriate and accurate content. AI tools can be used to create useful simulations and prompts that adapt to the input of the user, for example, on entering laboratory findings.^369^^,^^370^ Using AI assist in content generation reduces educator workload using the time gained to review outputs and undertake more productive tasks. AI tools can act as a virtual tutor, accessible 24/7, which again may enable the educator to focus on more complex tasks and needs of individual learners more effectively. AI tools can also be effectively deployed within simulated learning environments. For example, ChatGPT has been used to support training in the breaking of bad news by producing realistic scenarios and providing active roleplay with the user while proving clear feedback.^371^ Likewise, ChatGPT has been used within surgical rounds as a teaching tool to address gaps in knowledge, simulate difficult conversations, explore ethical challenges and support learners in building of differential diagnosis and decision making.^372^ Despite issues associated with the prompt design affecting results, accuracy of some outputs, and lack of specific references the use of the AI was seen as helpful. The use of AI-tools to assist medical students in clinical decision making demonstrated the advice produced was suitable and safe for students.^373^

Generative AI offers efficiency gain for academics in the generation of accessible teaching resources by reproducing content in multiple formats, generating alternative text of images and improving the quality and readability of language. With expert review of output, AI tools are also useful assistants in the design and generation of assessment items that are focused on pharmacology course content and to a standard appropriate for the learners’ stage on a program of study.^3^ The use of ChatGPT enabled the creation of 50 multiple choice questions in one tenth of the time take by University Professors to do the same with little to no difference reported in the appropriateness, clarity, specificity, relevance, discriminative power, or suitability of the questions.^374^ A study comparing human generated versus ChatGPT generated questions found that learners were unable to distinguish between to 2 with no significant differences in difficulty reported.^375^ There is therefore an efficiency gain, without compromise in quality, when AI tools are used to assist in the development of assessments.

As well as the development of assessments, the potential for AI tools in supporting marking and feedback processes is also being explored. There was found to be a strong correlation in the marking of short-answer questions by ChatGPT and a human assessor when provided with the same making rubric.^376^ Generative AI may also be helpful in providing support and feedback to learners during their studies. Indeed, feedback augmented by ChatGPT, prompting the AI to “make the original feedback constructive and encouraging,” was rated higher and strongly preferred by a cohort of university students over the human-generated original.^377^

AI tools are already being used by many learners to support their learning as a way to gathering and summarizing information, simplifying complex concepts, offering a personalized learning experience, practice scenarios and formative quizzes. AI-powered flashcard generators, such as Quizlet, Paperclips, and PDF2Anki, may rapidly convert content into flashcards supporting spaced repetition and improved memorization. However, the process of creating flashcards is in itself an important step for the process of learning with those who create their own flashcards outperforming those who rely on pregenerated equivalents.^378^ Although perceived by learners to be a useful learning assistant, AI tools may hallucinate, provide inaccurate or out-of-date information, and may lead learners to rely too heavily on the likes of ChatGPT potentially impacting their critical thinking and problem-solving skills.^379^^,^^380^ Despite these challenges, AI offers many opportunities to enhance student learning. The use of AI tools could assist the development of research skills and support discussion and debate for group and distance learning by providing a discussion structure, guidance, and real-time feedback to improve learner engagement.^379^

A survey of medical students found ChatGPT produced outputs that were considered to have greater clarity and organization than evidence-based sources, but the evidence-based sources were comprehensive.^381^ This suggests that many learners may be able to discern when it may be appropriate to use an AI tool and when it may be more appropriate to review the more comprehensive source of information. It is also worth remembering that learners have had access to the internet and search engines as a source of information for many years. Using Google to prepare for a test has been shown to improve performance by 11% over baseline. Interestingly using ChatGPT rather than Google to prepare for the test results in a similar improvement in test performance of 10% suggesting ChatGPT is as helpful but no better than using Google to prepare for a test.^382^

#### 6 Preparing graduates for an artificial intelligence–enabled world

AI technologies already have a number of applications in healthcare including in interpretation of diagnostic imaging, monitoring disease progression, and analyzing genetic data.^383^ The role of AI is also becoming more important in areas such as drug development, telemedicine, and robotic surgery.^384^ This highlights the need for educators to raise awareness, train students, and inspire the next generation of innovators by embedding AI within our course outcomes. In pharmacology specifically, AI is likely to impact many areas of practice, including research, drug discovery and development, therapeutic drug monitoring, and personalized healthcare.^385^, ^386^, ^387^ As an example, A GPT4-powered tool, ChemCrow, aims to streamline drug design and synthesis and has successfully executed the synthesis of an insect repellent from user inputs such as “plan and execute the synthesis of an insect repellent.”^388^

Although the rise of AI presents challenges and concerns, it also provides the opportunity to innovate to enhance pharmacology education. We have a responsibility to prepare our students for a future where AI is ubiquitous. AI literacy and supporting students to critically evaluate AI-generated content in the context of the capabilities and limitations of the tools uses should be embedded within curricula. By embracing AI, our students will gain the skills needed to thrive in this rapidly evolving landscape, we can support the integrity of assessments, as well as enhance the efficiency and effectiveness of educational practices.

## V Conclusions

Pharmacology education is undergoing a transformation, shaped by rapid scientific advances, evolving healthcare demands, and shifts in pedagogy and technology. As this review has illustrated, the discipline is no longer confined to traditional departmental boundaries or didactic teaching methods. Instead, it is increasingly embedded within interdisciplinary curricula, delivered through active and technology enhanced learning, and shaped by the diverse needs of a global student population. This evolution presents both opportunities and responsibilities, and we provide some top tips for navigating this complex landscape in Table 9.

**Table 9:** Top tips for educators

1. *Build education networks:* Get involved in local and international pharmacological societies’ education activities and initiatives (Table 3). 2. *Build your educational toolbox:* Engage with SoTL, DBER, and the pharmacology education literature to improve teaching practices and contribute to the broader academic community. 3. *Avoid curriculum overload:* Use evidence-based pharmacology curricula, content guides, and competency frameworks to select the most appropriate content (Table 4) 4. *Skills integration:* Incorporate skills students need to access, interpret, and use pharmacology knowledge independently, both as students and in their careers 5. *Continuous improvement:* Implement strategies for ongoing curriculum evaluation and refinement, using feedback from various stakeholders to adapt to evolving educational needs. 6. *Incorporate active learning strategies:* To enhance engagement and retention, embrace active, student-centered approaches (see section *Research-Informed Education – Constructivism and Active Learning*). 7. *Leverage technology where appropriate:* Utilise digital tools, simulations, and AI to support student- centered learning. 8. *Use authentic assessment:* Develop authentic assessments that reflect real-world tasks and skills, ensuring that evaluations are meaningful and relevant. 9. *Consider programmatic assessment:* Collect continuous data from various assessments to build a holistic picture of student competence. Provide regular, actionable feedback to support student development.

Educators must operate in an environment where foundational pharmacological knowledge must be preserved, even as pharmacology is integrated into broader biomedical and clinical contexts, and new competencies, ranging from digital literacy to interprofessional collaboration, become essential. They must act as advocates for the discipline, ensuring that core pharmacological principles and skills remain central to curricula across diverse programs. The development and dissemination of internationally agreed-upon core concepts and competency frameworks, such as those led by IUPHAR and other societies, are essential tools in this endeavor.

In integrated or interdisciplinary settings, it is especially important to maintain the visibility of pharmacology. Where standalone departments no longer exist, institutions should designate pharmacology leads to oversee curriculum content, ensure quality assurance, and advocate for the discipline in strategic planning. Without this, there is a risk of erosion in pharmacology expertise and a decline in graduate preparedness.

Looking ahead, pharmacology educators will play a central role in shaping how the discipline evolves. To do this effectively, they must be equipped not only with subject expertise but also with the tools, training, and institutional backing to lead curriculum development and implement active learning, authentic assessment, and appropriate use of digital tools including AI. National and international collaboration across institutions, societies, and regulatory bodies will be key to sharing best practices, aligning educational standards, and ensuring that pharmacology remains a clearly defined and well supported component of health and biomedical education. Pharmacology educators are encouraged to participate in networks such as IUPHAR-Ed and national education committees, not only to stay informed but to shape the future of the discipline.

## Conflicts of interest

The authors declare no conflicts of interest.

## References

[1] Buckingham J.C. (2012). Integrating pharmacology and clinical pharmacology in universities. Br J Clin Pharmacol.

[2] Freeman J. Student generative AI survey 2025. Higher Education Policy Institute. https://www.hepi.ac.uk/2025/02/26/student-generative-ai-survey-2025/.

[3] Sridharan K., Sequeira R.P. (2024). Artificial intelligence and medical education: application in classroom instruction and student assessment using a pharmacology & therapeutics case study. BMC Med Educ.

[4] Banerjee I., Robinson J., Annavarapu A., Gupta R. (2021). An insight of medical student’s preference and opinions to Pharmacology textbooks. J Biomed Sci.

[5] dos Reis Lívero F.A., da Silva G.R., Amaral E.C. (2021). Playfulness in the classroom: gamification favor the learning of pharmacology. Educ Inf Technol.

[6] Saadeh K., Henderson V., Paramasivam S.J., Jeevaratnam K. (2021). To what extent do preclinical veterinary students in the UK utilize online resources to study physiology. Adv Physiol Educ.

[7] Smith S.V. (2022). The COVID-19 pandemic and its effects on student performance in medical pharmacology. FASEB J.

[8] Singh C.K., Barme E., Ward R., Tupikina L., Santolini M. (2022). Quantifying the rise and fall of scientific fields. PLoS One.

[9] Lasagna L. (1969). The pharmaceutical revolution: its impact on science and society. Science.

[10] Nickles T, Zalta E.N., Nodelman U. (2024). The Stanford Encyclopedia of Philosophy (Spring 2024 Edition).

[11] Bynum W.F. Early History of the British pharmacological society. https://www.bps.ac.uk/getmedia/151d21c7-b759-4f60-bf90-d533f6eef321/Early-History-of-the-British-Pharmacological-Society.pdf.aspx.

[12] Lees P., Bäumer W., Toutain P.L. (2021). The decline and fall of materia medica and the rise of pharmacology and therapeutics in veterinary medicine. Front Vet Sci.

[13] Aronson J. (2009). When I use a word …materia medica, clinical pharmacology, and therapeutics. QJM Int J Med.

[14] Aronsson P., Booth S., Hägg S. (2015). The understanding of core pharmacological concepts among health care students in their final semester. BMC Med Educ.

[15] Habermann E.R. (1974). Rudolf Buchheim and the beginning of pharmacology as a science. Annu Rev Pharmacol Toxicol.

[16] Crowley F.C., Restini C., Burke K., Rieder M.J. (2025). Exploring the landscape of pharmacology education in health professions programs: from historical perspectives to current approaches to teaching. Eur J Pharmacol.

[17] Cuthbert A.W. (January 2006). A brief history of the British Pharmacological Society. Br J Pharmacol.

[18] Iacobelli T. Early 20th century reforms of medical education worldwide. Rockefeller Archive Center. https://resource.rockarch.org/story/early-20th-century-reforms-of-medical-education-worldwide/.

[19] Schmidt C.F. (1961). Pharmacology in a changing world. Annu Rev Physiol.

[20] Wagner R.R. (1962). The basic medical sciences, the revolution in biology and the future of medical education. Yale J Biol Med.

[21] Csáky T.Z. (1976). Is there an identity crisis in medical school pharmacology?. J Med Educ.

[22] Griesbacher T. Pharmacology departments world-wide. MedUni Vienna. https://meduni10.edis.at/pharma-www/indexdep.htm.

[23] Daniel K.L., McConnell M., Schuchardt A., Peffer M.E. (2022). Challenges facing interdisciplinary researchers: findings from a professional development workshop. PLoS One.

[24] Xu X., Hu J., Lyu X., Huang H., Cheng X. (2021). Exploring the interdisciplinary nature of precision medicine: network analysis and visualization. JMIR Med Inform.

[25] Fernald G.H., Capriotti E., Daneshjou R., Karczewski K.J., Altman R.B. (2011). Bioinformatics challenges for personalized medicine. Bioinformatics.

[26] Fasinu P.S., Wilborn T.W. (2024). Pharmacology education in the medical curriculum: challenges and opportunities for improvement. Pharmacol Res Perspect.

[27] Quesnelle K.M., Zaveri N.T., Schneid S.D. (2021). Design of a foundational sciences curriculum: applying the ICAP framework to pharmacology education in integrated medical curricula. Pharmacol Res Perspect.

[28] Pandit R., Gerrits M.A.F.M., Custers E.J.F.M. (2021). Assessing knowledge of pharmacokinetics in an integrated medical curriculum. Med Sci Educ.

[29] Gill M., Andersen E., Hilsmann N. (2019). Best practices for teaching pharmacology to undergraduate nursing students: a systematic review of the literature. Nurse Educ Today.

[30] Hughes G.J., Lee R., Sideras V. (2018). Design and delivery of clinical pharmacokinetics in colleges and schools of pharmacy. Am J Pharm Educ.

[31] Aronson J.K. (2012). Finding a VOICE for UK clinical pharmacology. Br J Clin Pharmacol.

[32] Sułkowski Ł., Fijałkowska J., Dzimińska M. (2019). Mergers in higher education institutions: a proposal of a novel conceptual model. Manag Fin.

[33] IQVIA IfHDS The global use of medicine in 2023 and outlook to 2027. https://www.iqvia.com/insights/the-iqvia-institute/reports-and-publications/reports/the-global-use-of-medicines-2023.

[34] Juneja J., Mai L., Albu N. The economic impact of the global pharmaceutical industry. https://www.wifor.com/en/download/economic-impact-of-the-global-pharmaceutical-industry/?wpdmdl=351721&refresh=673ca1ce6f5421732026830.

[35] Mendez K.M., Reinke S.N., Kelly R.S. (2025). A roadmap to precision medicine through post-genomic electronic medical records. Nat Commun.

[36] CMS CfMMS National health expenditure fact sheet. U.S. Department of Health & Human Services. https://www.cms.gov/data-research/statistics-trends-and-reports/national-health-expenditure-data/nhe-fact-sheet.

[37] NHS NBSA Prescription cost analysis, England 2022/23. https://www.nhsbsa.nhs.uk/statistical-collections/prescription-cost-analysis-england/prescription-cost-analysis-england-2022-23.

[38] Clews G. (2023). NHS drug costs in England rose to more than £19 billion in 2022/2023. Pharm J.

[39] Gerkens S., Lefèvre M., Bouckaert N. (2024).

[40] EFPIA The economic footprint of the pharmaceutical industry in Europe. https://www.efpia.eu/media/3dqjpl3x/economic-footprint-of-the-pharmaceutical-industry-in-europe-report.pdf.

[41] OECD/European Union Health at a glance: Europe 2020: state of health in the EU cycle. OECD Publishing, Paris. 10.1787/82129230-en.

[42] Dornan T., Ashcroft D., Heathfield H. (2009).

[43] World Health Organization (2024).

[44] Peck R. (2017). The pharmaceutical industry needs more clinical pharmacologists. Br J Clin Pharmacol.

[45] Ng Z.X., Yong P.H. (2022). The implication of multicultural education on students’ learning approaches in biosciences and pharmacy courses. J Appl Res Higher Educ.

[46] OECD. International student mobility. Organisation for Economic Co-operation and Development (OECD). https://www.oecd.org/en/data/indicators/international-student-mobility.html. Accessed August 30 2024.

[47] Drobne D. (2009). Toxicology has to use opportunities given by Bologna reform of higher education. Toxicol Lett.

[48] Nightcourses.com. Irish medical students in Poland at all-time high. https://www.nightcourses.com/blog/irish-medical-students-in-poland-at-all-time-high/#. Accessed August 26 2024.

[49] Nolan L. Why are there so many Irish vet students in Poland? RTÉ Newsroom. Accessed August 26, 2024. https://www.rte.ie/news/2023/0203/1353427-why-are-there-so-many-irish-vet-students-in-poland/.

[50] Stacey V. At least 8% of German medical students enrolled overseas. https://thepienews.com/at-least-8-of-german-medical-students-enrolled-overseas/.

[51] El Hadidi S. (2024). International branch campuses and pharmacy education in low-middle-income countries. Hosp Pharm.

[52] Advance HE Equality in higher education: statistical reports 2023. Advance HE. https://www.advance-he.ac.uk/knowledge-hub/equality-higher-education-statistical-reports-2023.

[53] UK Government Entry rates into higher education. UK Government. https://www.ethnicity-facts-figures.service.gov.uk/education-skills-and-training/higher-education/entry-rates-into-higher-education/latest/.

[54] Higher Education Statistics Agency Higher education student statistics: UK, 2022/23. https://www.hesa.ac.uk/news/08-08-2024/sb269-higher-education-student-statistics.

[55] Higher Education Student Numbers (UK Parliament) Higher education student statistics: UK, 2024/25. https://www.hesa.ac.uk/news/27-01-2026/sb273-higher-education-student-statistics.

[56] Statistics Netherlands An increasing number of Dutch people have completed higher education. https://www.cbs.nl/en-gb/news/2024/41/an-increasing-number-of-dutch-people-have-completed-higher-education.

[57] World Bank World development indicators. The World Bank Group. https://databank.worldbank.org/source/world-development-indicators.

[58] Britton J.D. (2021). Elaine; van der Erve, Laura. Which university degrees are best for intergenerational mobility?. https://ifs.org.uk/publications/which-university-degrees-are-best-intergenerational-mobility.

[59] Guilding C., Li Zhi P.K., Mohana Krishnan S., Hubbard P.S., McKeegan K.S. (2021). Insights into delivering cross-cultural medical education in the UK and Malaysia. Med Sci Educ.

[60] Long A., Ingram M., John Pugh W., Bowes P., Haigh S.J., Moss G. (2008). The effect of language background on teaching and learning in the master of pharmacy degree. Pharm Educ.

[61] British Pharmacological Society. Pharmacology education and employment landscape report launch. Accessed April 1, 2026. https://www.bps.ac.uk/news/blog/blog-article/?tx_news_pi1%5Baction%5D=detail&tx_news_pi1%5Bcontroller%5D=News&tx_news_pi1%5Bnews%5D=537&cHash=533a91e0a3492fd33476052c99fb5f94.

[62] Lloyd H., Hinton T., Bullock S. (2013). An evaluation of pharmacology curricula in Australian science and health-related degree programs. BMC Med Educ.

[63] De Baetselier E., Dijkstra N.E., Batalha L.M. (2024). Cross-sectional evaluation of pharmaceutical care competences in nurse education: how well do curricula prepare students of different educational levels?. BMC Nurs.

[64] Brinkman D.J., Tichelaar J., Okorie M. (2017). Pharmacology and therapeutics education in the European Union needs harmonization and modernization: a cross-sectional survey among 185 medical schools in 27 countries. Clin Pharmacol Ther.

[65] Tichelaar J., van Kan C., van Unen R.J. (2015). The effect of different levels of realism of context learning on the prescribing competencies of medical students during the clinical clerkship in internal medicine: an exploratory study. Eur J Clin Pharmacol.

[66] Winquist R.J., Mullane K., Williams M. (2014). The fall and rise of pharmacology–(re-) defining the discipline?. Biochem Pharmacol.

[67] Vallance P., Smart T.G. (2006). The future of pharmacology. Br J Pharmacol.

[68] Guilding C., Kelly-Laubscher R., White P. (2024). The future of pharmacology education: a global outlook. Expert Rev Clin Pharmacol.

[69] White P.J., Guilding C., Angelo T. (2023). Identifying the core concepts of pharmacology education: a global initiative. Br J Pharmacol.

[70] Achike F.I. (2010). Teaching pharmacology in an innovative medical curriculum: challenges of integration, technology, and future training. J Clin Pharmacol.

[71] Orme M., Sjöqvist F., Birkett D. Clinical Pharmacology in Health Care, Teaching and Research. Council for International Organizations of Medical Sciences (CIOMS). https://cioms.ch/publications/product/clinical-pharmacology-in-health-care-teaching-and-research/.

[72] Lundberg A., Stigmar M. (2024). Higher education teaching quality in the aftermath of the double disruption. Innov Higher Educ.

[73] Engels F. (2018). Pharmacology education: reflections and challenges. Eur J Pharmacol.

[74] Fukaya T., Nakamura D., Kitayama Y., Nakagoshi T. (2025). A systematic review and meta-analysis of research on mathematics and science pedagogical content knowledge: exploring its associations with teacher and student variables. Teach Teach Educ.

[75] Boyer E.L. (1990).

[76] Steiner H. What is SoTL? Kennesaw State University, Faculty Development and Recognition. https://facultydevelopment.kennesaw.edu/scholarly-teaching/news-articles/what-is-sotl.php.

[77] Kern B., Mettetal G., Dixson M., Morgan R.K. (2015). The role of SoTL in the academy: upon the 25th anniversary of Boyer’s Scholarship Reconsidered. J Scholarsh Teach Learn.

[78] Miller-Young J., Chick N.L. (2024).

[79] Babey A.-M., Koenig J., Cunningham M. (2025). Evaluating student understanding of core pharmacokinetic concepts. Eur J Pharmacol.

[80] Kelly-Laubscher R., Koenig J., Cunningham M. (2025). Evaluating student understanding of pharmacodynamics core concepts. Eur J Pharmacol.

[81] Forrest J., Elnaem M.H., Gleason S.E., Birnie C., Ryan M. (2022). White paper on the scholarship of teaching and learning: expanding the academic pharmacy promotion and tenure process. Pharm Educ.

[82] Harden R.M., Lilley P. (2018).

[83] Ouyang S., Zhang W., Xue L., Rashid A.M., Pyng H.S., Hassan A.B. (2025). The cultural compass: a systematic review on cultural dimensions theory in educational settings. Sage Open.

[84] Maxwell S, Walley T. (2003). BPS Clinical Section Committee. Teaching safe and effective prescribing in UK medical schools: a core curriculum for tomorrow's doctors.. Br J Clin Pharmacol.

[85] Brooks J.V., Dickinson B.L., Quesnelle K.M. (2023). Professional identity formation of basic science medical educators: a qualitative study of identity supports and threats. Acad Med.

[86] Griesbacher T. Societies of pharmacology world-wide. https://meduni10.edis.at/pharma-www/indexsoc.htm.

[87] El-Astal M. (2023). What is curriculum? Building a broader understanding of the term. J Curriculum Teach.

[88] AQF - Australian Qualifications Framework. AQF levels. Accessed March 2, 2025. https://www.aqf.edu.au/framework/aqf-levels#toc-aqf-level-6-criteria-3.

[89] QQI - Quality and Qualifications Ireland. National framework of qualifications. Accessed March 2, 2025. https://www.qqi.ie/what-we-do/the-qualifications-system/national-framework-of-qualifications.

[90] Quality Assurance Agency for Higher Education (2023) Subject Benchmark Statement: Biomedical Science and Biomedical Sciences. Gloucester: Quality Assurance Agency for Higher Education. Accessed March 13, 2026. https://www.qaa.ac.uk/the-quality-code/subject-benchmark-statements/subject-benchmark-statement-biomedical-science-and-biomedical-sciences

[91] EHEA - European Higher Education Area. EHEA members. Accessed March 2, 2025. https://ehea.info/page-members.

[92] GMC - General Medical Souncil. Outcomes for graduates. Accessed April 9, 2025. https://www.gmc-uk.org/education/standards-guidance-and-curricula/standards-and-outcomes/outcomes-for-graduates.

[93] NACNS - National Association of Clinical Nurse Specialists. Adult-gerontology clinical nurse specialist (CNS) competencies. Accessed April 9, 2025. https://nacns.org/resources/practice-and-cns-role/cns-competencies/adult-gero-competencies/, 2010.

[94] European Higher Education Area The framework of qualifications for the European Higher Education Area. https://ehea.info/media.ehea.info/file/WG_Frameworks_qualification/85/2/Framework_qualificationsforEHEA-May2005_587852.pdf.

[95] Guilding C., White P.J., Cunningham M. (2024). Defining and unpacking the core concepts of pharmacology: a global initiative. Br J Pharmacol.

[96] Salih S. (2016). Challenges of curriculum development for health sciences. BJESBS.

[97] MedicineWise N. (2021).

[98] Lim A.G., Honey M., Kilpatrick J. (2007). Framework for teaching pharmacology to prepare graduate nurse for prescribing in New Zealand. Nurse Educ Pract.

[99] Wallace M.J., Zecharia A., Guilding C., Tucker S., McFadzean I. (2021). Developing a new undergraduate pharmacology core curriculum: the British Pharmacological Society Delphi Method. Pharmacol Res Perspect.

[100] BPS - British Pharmacological Society. Undergraduate curriculum. Accessed March 16, 2026. https://www.bps.ac.uk/careers-education/teaching-pharmacology/undergraduate-curriculum/

[101] Ross S., Maxwell S. (2012). Prescribing and the core curriculum for tomorrow’s doctors: BPS curriculum in clinical pharmacology and prescribing for medical students. Br J Clin Pharmacol.

[102] Bailey S., Edmead C. (2023). Student-led approaches to introducing animal research for first year biomedical science and pharmacology undergraduates. Br J Pharmacol.

[103] BPS - British Pharmacological Society, Curriculum for the use of research animals. Accessed March 16, 2026. Use of research animals curriculum. https://www.bps.ac.uk/education-engagement/research-animals/curriculum-for-the-use-of-research-animals

[104] Lonsdale D.O., Guilding C., Koenig J., Okorie M., Sofat R., Maxwell S. (2026). Clinical Pharmacology and prescribing education: an updated medical school curriculum from the British Pharmacological Society. Br J Clin Pharmacol.

[105] Theobald R., Blumer J.B. (2023). The pharmacology knowledge objectives (KOs)-current status. J Pharmacol Exp Ther.

[106] ASPET - American Society for Pharmacology and Experimental Therapeutics. Pharmacology knowledge objectives. Accessed April 24, 2025. https://www.aspet.org/docs/default-source/dpe-division/pharmacology-ko-2024-update-07092024.pdf?sfvrsn=533773d3_0.

[107] Brinkman D.J., Tichelaar J., Mokkink L.B. (2018). Key learning outcomes for Clinical Pharmacology and therapeutics education in Europe: a modified Delphi Study. Clin Pharmacol Ther.

[108] EACPT Education Working Group. Accessed April 24, 2025. https://www.prescribingeducation.eu/.

[109] World Health Organization WHO model list of essential medicines – 23rd List. World Health Organization. https://www.who.int/publications/i/item/WHO-MHP-HPS-EML-2023.02.

[110] Core Concepts in Pharmacology. https://coreconceptspharmacology.org/.

[111] Donker E.M., Spitaleri Timpone P., Brinkman D.J. (2024). The European list of key medicines for medical education: a modified Delphi study. Clin Pharmacol Therap.

[112] BPS - British Pharmacological Society. Core skills—undergraduate pharmacology curriculum. Accessed March 16, 2026. https://www.bps.ac.uk/careers-education/teaching-pharmacology/undergraduate-curriculum/.

[113] RPS - Royal Pharmaceutical Society. A competency framework for all prescribers. Accessed April 24, 2025. https://www.rpharms.com/resources/frameworks/prescribers-competency-framework.

[114] Dijkstra N.E., De Baetselier E., Dilles T. (2021). Developing a competence framework for nurses in pharmaceutical care: a Delphi study. Nurse Educ Today.

[115] De Baetselier E., Van Rompaey B., Dijkstra N.E. (2021). The NUPHAC-EU framework for nurses’ role in interprofessional pharmaceutical care: cross-sectional evaluation in Europe. Int J Environ Res Public Health.

[116] Guilding C., Kelly-Laubscher R., Netere A. (2025). Developing an international concept-based curriculum for pharmacology education: the promise of core concepts and concept inventories. Br J Clin Pharmacol.

[117] Doria M.C.C. (2017). Outcomes-based approach to pharmacy curriculum review and redevelopment. Pharm Sci Asia.

[118] Aljuffali L., Faihan BinLebdah A., Alfaraj R. (2024). Closing the loop: strengthening course quality of Pharm.D. program via applying a comprehensive four-step review approach. Saudi Pharm J.

[119] Noble C., Shaw P.N., Nissen L., Coombes I., O’Brien M. (2011). Curriculum for uncertainty: certainty may not be the answer. Am J Pharm Educ.

[120] Click I., Lewis N.H., Karpa K. (2025). Assessment of individual disciplines before and after a shift to an active-learning and integrated curriculum. Br J Clin Pharmacol.

[121] Abdallah O., Ageeb R.A., Elkhalifa W.H.I. (2020). Evaluating prescribing competencies covered in a Canadian-accredited undergraduate pharmacy program in Qatar: a curriculum mapping process. BMC Med Educ.

[122] De Baetselier E., Dilles T., Feyen H., Haegdorens F., Mortelmans L., Van Rompaey B. (2022). Nurses’ responsibilities and tasks in pharmaceutical care: a scoping review. Nurs Open.

[123] Webb D.J. (2012). The roles of clinical pharmacologists in UK universities. Br J Clin Pharmacol.

[124] British Pharmacological Society Written evidence submitted by the British Pharmacological Society. 2021. HC 2021-22, RRE0054. https://committees.parliament.uk/writtenevidence/39694/pdf/.

[125] Jackson D. (2024).

[126] ASPET - American Society for Pharmacology and Experimental Therapeutics. ASPET strategic plan 2023–2027. Accessed April 9, 2025. https://www.aspet.org/docs/default-source/default-document-library/aspet-strategic-plan-2023-2027_v3.pdf.

[127] Seoane-Vazquez E., Rodriguez-Monguio R., Powers J.H. (2024). Analysis of US Food and Drug Administration new drug and biologic approvals, regulatory pathways, and review times, 1980–2022. Sci Rep.

[128] Eder J., Sedrani R., Wiesmann C. (2014). The discovery of first-in-class drugs: origins and evolution. Nat Rev Drug Discov.

[129] Park J.W., Lagniton P.N.P., Liu Y., Xu R.H. (2021). mRNA vaccines for COVID-19: what, why and how. Int J Biol Sci.

[130] Kenakin T., Christopoulos A. (2013). Signalling bias in new drug discovery: detection, quantification and therapeutic impact. Nat Rev Drug Discov.

[131] Ramzan I., Kahlaee R., Ramzan I. (2020). Biologics, Biosimilars, and Biobetters.

[132] Lakdawala N., Gronbeck C., Feng H. (2023). Comparison of prescribing patterns of non-physician clinicians and dermatologists in the Medicare population. Arch Dermatol Res.

[133] Maier C.B. (2019). Nurse prescribing of medicines in 13 European countries. Hum Resour Health.

[134] Baqir W., Miller D., Richardson G. (2012). A brief history of pharmacist prescribing in the UK. Eur J Hosp Pharm.

[135] Ecker S., Joshi R., Shanthosh J., Ma C., Webster R. (2020). Non-Medical prescribing policies: a global scoping review. Health Policy.

[136] McIntosh T., Stewart D., Forbes-McKay K., McCaig D., Cunningham S. (2016). Influences on prescribing decision-making among non-medical prescribers in the United Kingdom: systematic review. Fam Pract.

[137] GPhC - General Pharmaceutical Council. Standards for education and training of pharmacists. Accessed March 2, 2025. https://www.pharmacyregulation.org/students-and-trainees/education-and-training-providers/standards-education-and-training-pharmacists.

[138] Grimes T. New models of prescribing in the Republic of Ireland. https://imsn.ie/wp-content/uploads/2025/02/GrimesT.pdf.

[139] Russell C., Campion M., Grove M.E. (2024). Knowledge and attitudes on implementing cardiovascular pharmacogenomic testing. Clin Transl Sci.

[140] Just K.S., Steffens M., Swen J.J., Patrinos G.P., Guchelaar H.J., Stingl J.C. (2017). Medical education in pharmacogenomics-results from a survey on pharmacogenetic knowledge in healthcare professionals within the European pharmacogenomics clinical implementation project Ubiquitous Pharmacogenomics (U-PGx). Eur J Clin Pharmacol.

[141] Rahma A.T., Elsheik M., Ali B.R. (2020). Knowledge, attitudes, and perceived barriers toward genetic testing and pharmacogenomics among healthcare workers in the United Arab Emirates: a cross-sectional study. J Pers Med.

[142] Massingham L.J., Nuñez S., Bernstein J.A. (2022). 2022 Association of Professors of Human and Medical Genetics (APHMG) consensus-based update of the core competencies for undergraduate medical education in genetics and genomics. Genet Med.

[143] NHS England Initial education and training of pharmacists: genomic medicine indicative curriculum. https://www.hee.nhs.uk/sites/default/files/documents/NHS%20England%20Pharmacy%20Indicative%20Curriculum%20Genomics%20Mar%202025.pdf.

[144] Calzone K.A., Stokes L., Peterson C., Badzek L. (2024). Update to the essential genomic nursing competencies and outcome indicators. J Nurs Scholarsh.

[145] Gammal R.S., Lee Y.M., Petry N.J. (2022). Pharmacists leading the way to precision medicine: updates to the core pharmacist competencies in genomics. Am J Pharm Educ.

[146] Lairmore M.D., Ilkiw J. (2015). Animals used in research and education, 1966–2016: evolving attitudes, policies, and relationships. J Vet Med Educ.

[147] Freese T., Elzinga N., Heinemann M., Lerch M.M., Feringa B.L. (2024). The relevance of sustainable laboratory practices. RSC Sustain.

[148] Rai S., Sriram N., Alva P. (2024). Advancing green laboratory practices: a review of sustainability in healthcare. Int J Med Biochem.

[149] Badyal D.K., Desai C. (2014). Animal use in pharmacology education and research: the changing scenario. Indian J Pharmacol.

[150] Lawson R., Leymarie S., Nikitopoulos C. (2022). Alternative to animal experimentation in pharmacology teaching: development and validation of an equivalent digital learning tool. Pharmacol Res Perspect.

[151] British Pharmacological Society and the Physiological Society (2006). Tackling the need to teach integrative pharmacology and physiology: problems and ways forward. Trends Pharmacol Sci.

[152] In Vivo Pharmacology Training Group (2002). The fall and rise of in vivo pharmacology. Trends Pharmacol Sci.

[153] Seeley A., Corns L., Rouse J., Freestone N. Why should we use non-mammalian models for in vivo practical education? The Physiological Society. https://www.physoc.org/blog/why-should-we-use-non-mammalian-models-for-in-vivo-practical-education/.

[154] Seeley A., Bellamy C., Davies N.A., Wallace M.J. (2021). Lumbriculus variegatus: a novel organism for in vivo pharmacology education. Pharmacol Res Perspect.

[155] Carriere J.J., Davies N.A., Cunningham M.R., Wallace M.J., Seeley A. (2023). Co-created in vivo pharmacology practical classes using the novel organism Lumbriculus variegatus. Pharmacol Res Perspect.

[156] Schoenfeld T.J., Glenn N.O. (2022). Using Zebrafish embryos to study pharmacological effects on neural development in hands-on neurobiology laboratory activities. J Undergrad Neurosci Educ.

[157] Schmitz G.L., Nogara P.A., Medina N. (2022). Cockroaches: an alternative model to teach enzymatic inhibition to undergraduate students. J Biol Educ.

[158] Adedeji A.A., Vicente-Crespo M. (2017). Rejuvenating Research and Training in Biomedical Sciences in Nigeria: Drosophila Melanogaster as A Versatile Alternative Model. Arch Basic Appl Med.

[159] Royal Society of Biology Degree accreditation: important documents. https://www.rsb.org.uk/education/accreditation/Degree-Accreditation-Important-Documents.

[160] Institute of Biomedical Science IBMS accredited honours degrees. https://www.ibms.org/resources/documents/ibms-accredited-honours-degrees/.

[161] International Pharmaceutical Federation Green pharmacy practice: taking responsibility for the environmental impact of medicines. International Pharmaceutical Federation. https://www.fip.org/files/fip/publications/2015-12-Green-Pharmacy-Practice.pdf.

[162] International Pharmaceutical Federation FIP SustainabilityRx – A sustainable future for pharmacy, people and our planet. https://sustainability.fip.org/.

[163] Banning M. (2003). Pharmacology education: a theoretical framework of applied pharmacology and therapeutics. Nurse Educ Today.

[164] Baldwin M.J., Abouyannis M., Butt T.F. (2012). Essential therapeutics skills required of junior doctors. Perspect Med Educ.

[165] Richir M.C., Tichelaar J., Geijteman E.C.T., de Vries T.P.G.M. (2008). Teaching clinical pharmacology and therapeutics with an emphasis on the therapeutic reasoning of undergraduate medical students. Eur J Clin Pharmacol.

[166] Fajt V.R. (2008). Skills and competencies required by veterinary pharmacologists: a blueprint for graduate education in veterinary pharmacology in North America. J Vet Pharmacol Ther.

[167] Rangachari P.K. (2003). Poised between the pedantic and the puerile: physicians-to-be in a problem-based learning program. Biochem Mol Biol Educ.

[168] White P.J., Davis E.A., Santiago M. (2021). Identifying the core concepts of pharmacology education. Pharmacol Res Perspect.

[169] Swords C.M., Porter J.S., Hawkins A.J. (2023). Science communication training imparts confidence and influences public engagement activity. J Microbiol Biol Educ.

[170] Rubaiy H.N. (2021). Strategies to inspire students’ engagement in pharmacology courses. Pharmacy.

[171] Candler C., Ihnat M., Huang G. (2007). Pharmacology education in undergraduate and graduate medical education in the United States. Clin Pharmacol Ther.

[172] Brinkman D.J., Tichelaar J., Schutte T. (2017). Essential competencies in prescribing: a first European cross-sectional study among 895 final-year medical students. Clin Pharmacol Ther.

[173] General Medical Council Outcomes for graduates. https://www.gmc-uk.org/education/standards-guidance-and-curricula/standards-and-outcomes/outcomes-for-graduates/outcomes-for-graduates.

[174] Magavern E.F., Hitchings A., Bollington L. (2024). UK Prescribing Safety Assessment (PSA): the development, implementation and outcomes of a national online prescribing assessment. Br J Clin Pharmacol.

[175] Maxwell S.R.J., Coleman J.J., Bollington L., Taylor C., Webb D.J. (2017). Prescribing Safety Assessment 2016: delivery of a national prescribing assessment to 7343 UK final-year medical students. Br J Clin Pharmacol.

[176] Karpa K.D., Hom L.L., Huffman P. (2015). Medication safety curriculum: enhancing skills and changing behaviors. BMC Med Educ.

[177] Donker E.M., Brinkman D.J., van Rosse F. (2022). Do we become better prescribers after graduation: a 1-year international follow-up study among junior doctors. Br J Clin Pharmacol.

[178] Vygotsky L.S. (1987).

[179] Bada S.O., Olusegun S. (2015). Constructivism learning theory: a paradigm for teaching and learning. J Res Method Educ.

[180] Michael J. (2006). Where’s the evidence that active learning works?. Adv Physiol Educ.

[181] Michael J., Modell H.I. (2003).

[182] Wieman C., Perkins K. (2005). Transforming physics education. Phys Today.

[183] Arons A.B., Holbrow C. (1990).

[184] Hake R.R. (1998). Interactive-engagement versus traditional methods: a six-thousand-student survey of mechanics test data for introductory physics courses. Am J Phys.

[185] Wieman C. (2017).

[186] Deslauriers L., Schelew E., Wieman C. (2011). Improved learning in a large-enrollment physics class. Science.

[187] Crouch C.H., Mazur E. (2001). Peer Instruction: ten years of experience and results. Am J Phys.

[188] Dancy M., Henderson C., Apkarian N. (2024). Physics instructors’ knowledge and use of active learning has increased over the last decade but most still lecture too much. Phys Rev Phys Educ Res.

[189] Brewer C.A., Smith D. (2011).

[190] Ledbetter M.L.S. (2012). Vision and change in undergraduate biology education: a call to action presentation to Faculty for Undergraduate Neuroscience. J Undergrad Neurosci Educ.

[191] Freeman S., Eddy S.L., McDonough M. (2014). Active learning increases student performance in science, engineering, and mathematics. Proc Natl Acad Sci U S A.

[192] McLellan L., Tully M.P., Dornan T. (2012). How could undergraduate education prepare new graduates to be safer prescribers?. Br J Clin Pharmacol.

[193] Donker E.M., Osmani H., Brinkman D.J. (2023). The impact of a summative national prescribing assessment and curriculum type on the development of the prescribing competence of junior doctors. Eur J Clin Pharmacol.

[194] Brinkman D.J., Monteiro T., Monteiro E.C., Richir M.C., van Agtmael M.A., Tichelaar J. (2021). Switching from a traditional undergraduate programme in (clinical) pharmacology and therapeutics to a problem-based learning programme. Eur J Clin Pharmacol.

[195] White P.J., Naidu S., Yuriev E., Short J.L., McLaughlin J.E., Larson I.C. (2017). Student engagement with a flipped classroom teaching design affects pharmacology examination performance in a manner dependent on question type. Am J Pharm Educ.

[196] White P.J., Larson I., Styles K. (2016). Adopting an active learning approach to teaching in a research-intensive higher education context transformed staff teaching attitudes and behaviours. Higher Educ Res Dev.

[197] Gregory M.S.-J., Lodge J.M. (2015). Academic workload: the silent barrier to the implementation of technology-enhanced learning strategies in higher education. Distance Educ.

[198] Wain A. (2017). Learning through reflection. Br J Midwif.

[199] Austin Z., Duncan-Hewitt W.C. (2005). Faculty, student, and practitioner development within a community of practice. Am J Pharm Educ.

[200] Sumanasekera W., Turner C., Ly K., Hoang P., Jent T., Sumanasekera T. (2020). Evaluation of multiple active learning strategies in a pharmacology course. Curr Pharm Teach Learn.

[201] Tripathi R.K., Sarkate P.V., Jalgaonkar S.V., Rege N.N. (2015). Development of active learning modules in pharmacology for small group teaching. Educ Health.

[202] Carstensen S.S., Kjaer C., Möller S., Bloksgaard M. (2020). Implementing collaborative, active learning using peer instructions in pharmacology teaching increases students’ learning and thereby exam performance. Eur J Pharmacol.

[203] Kennedy D.R. (2019). Redesigning a pharmacology course to promote active learning. Am J Pharm Educ.

[204] Yanagita T., Kanaoka M., Kinoshita Y., Takeya R. (2022). Nursing pharmacology education and active-learning. Nihon Yakurigaku Zasshi.

[205] Gorman L. (2017). Promoting the active learning of pharmacology and clinical therapeutics utilizing team-based learning (TBL) methods in second year systems modules. FASEB J.

[206] Yiin S.J., Chern C.L. (2023). The effects of an active learning mechanism on cognitive load and learning achievement: a new approach for pharmacology teaching to Taiwanese nursing students. Nurse Educ Today.

[207] Guilding C., Pye R.E., Butler S., Atkinson M., Field E. (2021). Answering questions in a co-created formative exam question bank improves summative exam performance, while students perceive benefits from answering, authoring, and peer discussion: a mixed methods analysis of PeerWise. Pharmacol Res Perspect.

[208] Baños J.E., Blanco-Reina E., Bellido-Estévez I. (2024). Beyond lectures and practical courses: teaching pharmacology using imaginative pedagogical tools. Pharmacol Res.

[209] Baños J.-E., Lucena M.I., Farré M. (2019). The usefulness of TV medical dramas for teaching clinical pharmacology: a content analysis of House, MD. Educ Med.

[210] Liu L., Du X., Zhang Z., Zhou J. (2019). Effect of problem-based learning in pharmacology education: a meta-analysis. Stud Educ Eval.

[211] Quesnelle K.M., Bright D.R., Salvati L.A. (2018). Interprofessional education through a telehealth team based learning exercise focused on pharmacogenomics. Curr Pharm Teach Learn.

[212] Rangachari P.K. (2011). Steps to pluripotent learning: provocative teaching. Adv Physiol Educ.

[213] Xiao C.L., Ren H., Chen H.Q. (2023). Multidimensional evaluation of teaching strategies for pharmacology based on a comprehensive analysis involving 21,269 students. Front Pharmacol.

[214] Michaelsen L.K., Knight A.B., Fink L.D. (2023).

[215] Whitley H.P., Bell E., Eng M. (2015). Practical team-based learning from planning to implementation. Am J Pharm Educ.

[216] Carrasco G.A., Behling K.C., Gentile M., Fischer B.D., Ferraro T.N. (2021). Effectiveness of a Team-Based Learning exercise in the learning outcomes of a medical pharmacology course: insight from struggling students. Naunyn Schmiedebergs Arch Pharmacol.

[217] Kim D.H., Lee J.H., Kim S.A. (2020). The pharmacology course for preclinical students using team-based learning. Korean J Med Educ.

[218] Dunaway G.A. (2005). Adaption of team learning to an introductory graduate pharmacology course. Teach Learn Med.

[219] Zgheib N.K., Simaan J.A., Sabra R. (2010). Using team-based learning to teach pharmacology to second year medical students improves student performance. Med Teach.

[220] Hashilkar N., Getula M., Ameen A. (2014). Effectiveness of team based learning to teach pharmacology for phase-II MBBS students. J Med Sci.

[221] Mehnaatamai Mohanram A., Zhong Q. (2014). Assessing team-based learning method's effectiveness in medical pharmacology teaching (719.8). FASEB J.

[222] Attia R.T., Mandour A.A. (2023). Team-based learning-adopted strategy in pharmacy education: pharmacology and medicinal chemistry students’ perceptions. Future J Pharm Sci.

[223] Chen D., Yue H., Liu S., Meng L., Yin W. (2022). The introduction of team-based learning into the clinical pharmacology section of the endodontics clinical course. Clin Exp Pharmacol Physiol.

[224] El-Banna M.M., Whitlow M., McNelis A.M. (2020). Improving pharmacology standardized test and final examination scores through team-based learning. Nurse Educ.

[225] Alizadeh M., Masoomi R., Mafinejad M.K., Parmelee D., Khalaf R.J., Norouzi A. (2024). Team-based learning in health professions education: an umbrella review. BMC Med Educ.

[226] Nguyen T., Wong E., Pham A. (2016). Incorporating team-based learning into a physician assistant Clinical Pharmacology course. J Physician Assist Educ.

[227] McCormack N., Ryan M. (2022). Compendium of Active Learning & Assessment for Student Engagement-Volume 2: TUS-MMW.

[228] Korayem G.B., Alghamdi A.A., Aljuhani O., Ivy D., Alhubaishi A.A., Alkofide H. (2024). Team-based learning versus traditional teaching effect on pharmacy Students’ Performance: a systematic review and Meta-Analysis. Saudi Pharm J.

[229] Guilding C., Hardisty J., Randles E. (2020/10/13). Designing and evaluating an interprofessional education conference approach to antimicrobial education. BMC Med Educ.

[230] Burgess A., Kalman E., Haq I., Leaver A., Roberts C., Bleasel J. (2020). Interprofessional team-based learning (TBL): how do students engage?. BMC Med Educ.

[231] Barrows H.S. (1986). A taxonomy of problem-based learning methods. Med Educ.

[232] Hmelo-Silver C.E. (2004). Problem-based learning: what and how do students learn?. Educ Psychol Rev.

[233] Ren S., Li Y., Pu L., Feng Y. (2023). Effects of problem-based learning on delivering medical and nursing education: a systematic review and meta-analysis of randomized controlled trials. Worldviews Evid-Based Nurs.

[234] Li T., Wang W., Li Z., Wang H., Liu X. (2022). Problem-based or lecture-based learning, old topic in the new field: a meta-analysis on the effects of PBL teaching method in Chinese standardized residency training. BMC Med Educ.

[235] Dochy F., Segers M., Van den Bossche P., Gijbels D. (2003). Effects of problem-based learning: a meta-analysis. Learn Instruction.

[236] Norman G.R., Schmidt H.G. (1992). The psychological basis of problem-based learning: a review of the evidence. Acad Med.

[237] Schmidt H.G., Rotgans J.I., Yew E.H.J. (2011). The process of problem-based learning: what works and why. Med Educ.

[238] Chian M.M., Bridges S.M., Lo E.C.M. (2019). The triple jump in problem-based learning: unpacking principles and practices in designing assessment for curriculum alignment. Interdiscip J Problem-Based Learn.

[239] Feletti G., Ryan G. (1994). The triple jump exercise in inquiry-based learning: a case study showing directions for further research. Assess Eval Higher Educ.

[240] Vernon D.T., Blake R.L. (1993). Does problem-based learning work? A meta-analysis of evaluative research. Acad Med.

[241] Dolmans D.H.J.M., Loyens S.M.M., Marcq H., Gijbels D. (2016). Deep and surface learning in problem-based learning: a review of the literature. Adv Health Sci Educ Theory Pract.

[242] Matsuda Y., Falcon A., Porter A. (2024). Implementation of problem-based learning modules in an introduction to public health course. Front Public Health.

[243] Anthony L., A R., T I., Banerjee S. (2025). Comparison of problem-based learning and didactic lecture as a teaching–learning method among undergraduate medical students: an interventional study. J Pharmacol Pharmacother.

[244] Yang X., Yang Z., Ma S., Yan M., Yang Y. (2024). Evaluation of problem-based learning for pharmacology based on a comprehensive analysis in undergraduate students. Medicine.

[245] Wood D.F. (2003). Problem based learning. BMJ.

[246] Pierce B., van de Mortel T., Allen J., Mitchell C. (2024). The influence of near-peer teaching on undergraduate health professional students’ self-efficacy beliefs: a systematic integrative review. Nurse Educ Today.

[247] Brierley C., Ellis L., Reid E.R. (2022). Peer-assisted learning in medical education: a systematic review and meta-analysis. Med Educ.

[248] Demak I.P.K., Tanra A.A.M., Syamsi N., Nur R., Wahyuni R.D. (2021). Learning pharmacology through peer tutoring. Gac Sanit.

[249] Mohammed Z., Shah M.S., Abbas I. (2025). Pharmacist peer-led teaching enhances medical undergraduate prescribing: a mixed-methods study. Clin Teach.

[250] Azer SA (2001). Problem-based learning. Challenges, barriers and outcome issues. Saudi Med J.

[251] Kaufman D.M. (2003). Applying educational theory in practice. BMJ.

[252] Reddy P. (2022). Group work in undergraduate research: turning bane into boon. Afr J Inter Multidiscip Stud.

[253] Huerta M.V., Sajadi S., Schibelius L., Ryan O.J., Fisher M. (2024). An exploration of psychological safety and conflict in first-year engineering student teams. J Eng Educ.

[254] Albanese M.A., Mitchell S. (1993). Problem-based learning: a review of literature on its outcomes and implementation issues. Acad Med.

[255] van der Vleuten C.P.M., Schuwirth L.W.T. (2019). Assessment in the context of problem-based learning. Adv Health Sci Educ Theory Pract.

[256] Gijbels D., Dochy F., Van den Bossche P., Segers M. (2005). Effects of problem-based learning: a meta-analysis from the angle of assessment. Rev Educ Res.

[257] Amin I., Tamang E.L., Khan M. (2023). Effectiveness of problem-based learning versus traditional lecture method in terms of knowledge among nursing students in a selected nursing college of Kashmir. Indian J Contin Nurs Educ.

[258] Steinert Y. (2000/01/01). Faculty development in the new millennium: key challenges and future directions. Med Teach.

[259] Burgess A., van Diggele C., Mellis C. (2018). Mentorship in the health professions: a review. Clin Teach.

[260] Branch C., Gill T., Apampa B. (2015). Can you learn from a dummy? Pharmacy students’ views and perceptions of SimMan, a human patient simulator. Pharm Educ.

[261] Swamy M., Sawdon M., Chaytor A., Cox D., Barbaro-Brown J., McLachlan J. (2014). A study to investigate the effectiveness of SimMan® as an adjunct in teaching preclinical skills to medical students. BMC Med Educ.

[262] Guilding C. (2016). Choose your own story: combining interactive voting technology and high-fidelity patient simulations in the lecture theatre, for large group preclinical medical education. BMJ Simul Technol Enhanc Learn.

[263] Seybert A.L., Laughlin K.K., Benedict N.J., Barton C.M., Rea R.S. (2006). Pharmacy student response to patient-simulation mannequins to teach performance-based pharmacotherapeutics. Am J Pharm Educ.

[264] Andersen P., Cox K. (2020). SimMan 3G™: manikin-led simulation orientation. Clin Simul Nurs.

[265] Dempster J. Strathclyde pharmacology simulations. https://spider.science.strath.ac.uk/sipbs/page.php?page=software_sims.

[266] Ara T., Kitamura H. (2025). Review of the simulators used in pharmacology education and statistical models when creating the simulators. Appl Biosci.

[267] Zhang T., Tyson J.J. (2022). Understanding virtual patients efficiently and rigorously by combining machine learning with dynamical modelling. J Pharmacokinet Pharmacodyn.

[268] Jumper J., Evans R., Pritzel A. (2021). Highly accurate protein structure prediction with AlphaFold. Nature.

[269] Tolentino R., Baradaran A., Gore G., Pluye P., Abbasgholizadeh-Rahimi S. (2024). Curriculum frameworks and educational programs in AI for medical students, residents, and practicing physicians: scoping review. JMIR Med Educ.

[270] Ng F.Y.C., Thirunavukarasu A.J., Cheng H. (2023). Artificial intelligence education: an evidence-based medicine approach for consumers, translators, and developers. Cell Rep Med.

[271] Lu W., Zhang J., Huang W. (2024/02/05). DynamicBind: predicting ligand-specific protein-ligand complex structure with a deep equivariant generative model. Nat Commun.

[272] Karelina M., Noh J.J., Dror R.O. (2023). How accurately can one predict drug binding modes using AlphaFold models?. eLife.

[273] Kim K., Xie N., Hammersmith L., Berrocal Y., Roni M.A. (2023). Impact of virtual reality on pharmacology education: a pilot study. Cureus.

[274] Dechsling A., Vister O.M., Johansen T.E., Børtveit L., Herikstad Y., Nordahl-Hansen A. (2024). Implementing virtual reality in special education: teachers’ perspectives. Int J Disabil Dev Educ.

[275] Mirzaian E., Franson K.L. (2021). Leading a digital transformation in pharmacy education with a pandemic as the accelerant. Pharmacy.

[276] Hafner S., Zolk O., Barth H. (2022). COVID-19 pandemic-related adaptations of medical education in clinical pharmacology—impact on students and lecturers at a German university. Naunyn Schmiedebergs Arch Pharmacol.

[277] Azamam N.N., Suratman S., Mustaffa M.F., Ramli N.A., Maniam S., Ali A.A. (2022). E-learning in pharmacology education during COVID 19 pandemic: students’ preference & perception of assessments. Malays J Med Health Sci.

[278] Fuller K.A., Heldenbrand S.D., Smith M.D., Malcom D.R. (2020). A paradigm shift in US experiential pharmacy education accelerated by the COVID-19 pandemic. Am J Pharm Educ.

[279] Sakr F., Fahs I., Dimassi A. (2022). Experiential pharmacy education in trying times: lessons learned from the COVID-19 pandemic. Pharm Educ.

[280] Rhoney D.H., Singleton S., Nelson N.R., Anderson S.M., Hubal R. (2021). Forces driving change in pharmacy education: opportunities to take academic, social, technological, economic, and political into the future. J Am Coll Clin Pharm.

[281] Hammoudi Halat D., Younes S., Safwan J., Akiki Z., Akel M., Rahal M. (2022). Pharmacy students’ mental health and resilience in COVID-19: an assessment after one year of online education. Eur J Investig Health Psychol Educ.

[282] Vaskivska H.O., Palamar S.P., Kravtsova N.V., Khodakivska O.V. (2021). Transformation of the learning process in higher education institutions under the influence of the pandemic COVID-19. Wiad Lek.

[283] Karara A.H., Nan A., Goldberg B., Shukla R. (2021). Use of science lab simulation during a two-week virtual biomedical research training summer camp for underserved minority youth: a COVID-19 adjustment. J Stem Outreach.

[284] Tsirulnikov D., Suart C., Abdullah R., Vulcu F., Mullarkey C.E. (2023). Game on: immersive virtual laboratory simulation improves student learning outcomes & motivation. FEBS Open Bio.

[285] Wilhelmus M.M.M., Drukarch B. (2025). Hands-on practicals in pharmacology teaching at university level: outpaced by computer-based simulations, or is there a (blended) future?. Eur J Pharmacol.

[286] Delage C., Palayer M., Lerouet D., Besson V.C. (2024). ‘Pharmacotrophy’: a playful tournament for game- and team-based learning in pharmacology education—assessing its impact on students’ performance. BMC Med Educ.

[287] MacKenzie I., Parsons K., Lee Y.P. (2024). Escape rooms in pharmacy education: more than just a game. Curr Pharm Teach Learn.

[288] El-Sabagh H.A. (2021). Adaptive e-learning environment based on learning styles and its impact on development students’ engagement. Int J Educ Technol Higher Educ.

[289] Bzowyckyj A.S., Blake E., Crabtree B. (2021). Advancing pharmacy education and workforce development amid the COVID-19 pandemic: report of the 2020–2021 AACP academic affairs committee. Am J Pharm Educ.

[290] Knight P.T. (2002). Summative assessment in higher education: practices in disarray. Stud Higher Educ.

[291] Schellekens L.H., Bok H.G.J., de Jong L.H., van der Schaaf M.F., Kremer W.D.J., van der Vleuten C.P.M. (2021). A scoping review on the notions of Assessment as Learning (AaL), Assessment for Learning (AfL), and Assessment of Learning (AoL). Stud Educ Eval.

[292] Serrano Santos J.M. (2017). 3rd International Conference on Higher Education Advances, HEAd’17.

[293] Lee C.Y., Miller C., Bone E., Kusljic S. (2025). Connecting nursing cohorts in authentic learning activities through a redesigned pharmacology curriculum. Teach Learn Nurs.

[294] Lavanya S.H., Kalpana L., Veena R.M., Bharath Kumar V.D. (2016). Role-play as an educational tool in medication communication skills: students’ perspectives. Indian J Pharmacol.

[295] Dewhurst D., Ward R. (2014). The virtual pharmacology lab — a repository of free educational resources to support animal-free pharmacology teaching. Altern Lab Anim.

[296] Rangachari P.K. (2002). Student-designed clinical trials: evaluating self-directed learning in pharmacology. Naunyn Schmiedebergs Arch Pharmacol.

[297] Schramm G.E., Narayanan P.P., Chutka D.S., Nicholson W.T. (2017). Implementation of an interprofessional clinical pharmacology selective learning experience for pharmacy residents and medical students. Am J Health Syst Pharm.

[298] French S., Dickerson A., Mulder R.A. (2024). A review of the benefits and drawbacks of high-stakes final examinations in higher education. Higher Educ.

[299] Peeters M.J., Cor M.K. (2020). Guidance for high-stakes testing within pharmacy educational assessment. Curr Pharm Teach Learn.

[300] Gérard A.O., Merino D., Labriffe M. (2025). Evaluating and leveraging large language models in clinical pharmacology and therapeutics assessment: from exam takers to exam shapers. Br J Clin Pharmacol.

[301] Pignatelli-Espejo A., Kelly-Laubscher R., Barry Ó.P. (2025). Exploring the knowledge demands of a pharmacology assessment using Legitimation Code Theory. Eur J Pharmacol.

[302] Martin R.D., Naziruddin Z. (2020). Systematic review of student anxiety and performance during objective structured clinical examinations. Curr Pharm Teach Learn.

[303] Hadi M.A., Ali M., Haseeb A., Mohamed M.M.A., Elrggal M.E., Cheema E. (2018). Impact of test anxiety on pharmacy students’ performance in Objective Structured Clinical Examination: a cross-sectional survey. Int J Pharm Pract.

[304] Robinson P., Morton L., Haran H., Manton R. (2017). Mock OSCEs improve medical students’ confidence and reduce anxiety related to summative examinations. EIMJ.

[305] Corwin L.A., Prunuske A., Seidel S.B. (2018). Scientific presenting: using evidence-based classroom practices to deliver effective conference presentations. CBE Life Sci Educ.

[306] Meng X., Yang L., Sun H., Du X., Yang B., Guo H. (2019). Using a novel student-centered teaching method to improve pharmacy student learning. Am J Pharm Educ.

[307] Schuwirth L., van der Vleuten C., Durning S.J. (2017). What programmatic assessment in medical education can learn from healthcare. Perspect Med Educ.

[308] van der Vleuten C., Lindemann I., Schmidt L. (2018). Programmatic assessment: the process, rationale and evidence for modern evaluation approaches in medical education. Med J Aust.

[309] Schut S., Maggio L.A., Heeneman S., van Tartwijk J., van der Vleuten C., Driessen E. (2021). Where the rubber meets the road—an integrative review of programmatic assessment in health care professions education. Perspect Med Educ.

[310] Govaerts M., Van der Vleuten C., Schut S. (2022). Implementation of programmatic assessment: challenges and lessons learned. Educ Sci.

[311] Yan Z., Yang L. (2021). Assessment as Learning.

[312] Lakhtakia R., Otaki F., Alsuwaidi L., Zary N. (2022). Assessment as learning in medical education: feasibility and perceived impact of student-generated formative assessments. JMIR Med Educ.

[313] Yeung M.A., Lam P., McNaught C. (2008). Student-creation of eCases for clinical reasoning in pharmacy. Australas J Peer Learn.

[314] Enslein T., Kosack E., Wetzel H.N. (2023). Student perceptions of scientific writing in pharmacology: student generation of collaborative rubrics to score a social pharmacology writing project. Pharmacol Res Perspect.

[315] Davis L.E. (2014). A workshop series using peer-grading to build drug information, writing, critical-thinking, and constructive feedback skills. Am J Pharm Educ.

[316] Yusuff K.B. (2015). Does self-reflection and peer-assessment improve Saudi pharmacy students’ academic performance and metacognitive skills?. Saudi Pharm J.

[317] Wiggins G. (1990). The case for authentic assessment. Pract Assess Res Eval.

[318] Miller E., Konstantinou I. (2022). Using reflective, authentic assessments to embed employability skills in higher education. J Work-Appl Manag.

[319] Van K., Tasawar S., Brendel E.B.K. (2025). Using a “Students as Partners” model to develop an authentic assessment promoting employability skills in undergraduate life science education. FEBS Open Bio.

[320] Maharajan M.K., Sivapalan S., Rajiah K. (2025). Empowering students in curriculum design and pedagogy: perceptions of pharmacy students as partners; A qualitative study. Curr Pharm Teach Learn.

[321] Lim A.S., Ling Y.L., Wilby K.J., Mak V. (2024). What’s been trending with OSCEs in pharmacy education over the last 20 years? A bibliometric review and content analysis. Curr Pharm Teach Learn.

[322] Laura K. (2024). Preparing for OSCEs in pharmacy training. Pharm J.

[323] Badyal D.K. (2024). Objective structured practical examination in pharmacology MBBS assessment. Natl J Pharmacol Ther.

[324] Shenoy P.J., Kamath P., Sayeli V., Pai S. (2017). Standardization and validation of objective structured practical examination in pharmacology: our experience and lessons learned. Indian J Pharmacol.

[325] Karunaratne N., Exintaris B., Zhou E., Pyun J., Budzyn K., Lim A. (2024). “A pilot trial of objective structured practical examinations (OSPEs) in non-vocational science-based degrees. Innov Educ Teach Int.

[326] Hennig S., Staatz C.E., Bond J.A., Leung D., Singleton J. (2019). Quizzing for success: evaluation of the impact of feedback quizzes on the experiences and academic performance of undergraduate students in two clinical pharmacokinetics courses. Curr Pharm Teach Learn.

[327] Nydia R.H. (2002). Effective use of a range of authentic assessments in a web assisted pharmacology course. J Educ Technol Soc.

[328] Cuddy P., Oki J., Wooten J. (2001). Online peer evaluation in basic pharmacology. Acad Med.

[329] Berger D.J., Nickolich S., Nasir M. (2024). Introduction to tobacco cessation and motivational interviewing: evaluation of a lecture and case-based learning activity for medical students. Cureus.

[330] Bettonte S., Berton M., Battegay M., Stader F., Marzolini C. (2024). Development of a physiologically-based pharmacokinetic model to simulate the pharmacokinetics of intramuscular antiretroviral drugs. CPT Pharmacometrics Syst Pharmacol.

[331] Dewhurst D. (2008). Is it possible to meet the learning objectives of undergraduate pharmacology classes with non-animal models. AATEX.

[332] Biggs J. (1996). Enhancing teaching through constructive alignment. Higher Educ.

[333] Sotiriadou P., Logan D., Daly A., Guest R. (2020). The role of authentic assessment to preserve academic integrity and promote skill development and employability. Stud Higher Educ.

[334] Sakzad A., Paul D., Sheard J. (2024). Proceedings of the 55th ACM Technical Symposium on Computer Science Education.

[335] Thornhill-Miller B., Camarda A., Mercier M. (2023). Creativity, critical thinking, communication, and collaboration: assessment, certification, and promotion of 21st century skills for the future of work and education. J Intell.

[336] Rayamajhi S., Machin A., Breen C., Gebreheat G., Paterson R. (2024). Quality and impact of pharmacology digital simulation education on pre-registration healthcare students: a systematic literature review. Nurse Educ Today.

[337] Charles K.A., Pairman L., Moon E. (2025). The impact of a preprescribing formative assessment on learning in final-year medical students using hospital inpatient electronic prescribing systems. Br J Clin Pharmacol.

[338] Brinkman D.J., Donker E.M., Tichelaar J. (2025). Prescribing competence: the pros and cons of different methods for assessment. Br J Clin Pharmacol.

[339] Maxwell S.R.J., Cameron I.T., Webb D.J. (2015). Prescribing safety: ensuring that new graduates are prepared. Lancet.

[340] Maxwell S.R.J., Webb D.J. (2019). Improving medication safety: focus on prescribers and systems. Lancet.

[341] Hardisty J., Davison K., Statham L., Fleming G., Bollington L., Maxwell S. (2018). Exploring the utility of the Prescribing Safety Assessment in pharmacy education in England: experiences of pre-registration trainees and undergraduate (MPharm) pharmacy students. Int J Pharm Pract.

[342] Jansen D.R.M., Keijsers C.J.P.W., Kornelissen M.O., Olde Rikkert M.G.M., Kramers C., (on behalf of the education working group of the Dutch Society for Clinical Pharmacology and Biopharmacy) (2019). Towards a “prescribing license” for medical students: development and quality evaluation of an assessment for safe prescribing. Eur J Clin Pharmacol.

[343] Donker E.M., Brinkman D.J., Richir M.C. (2022). The European Prescribing Exam: assessing whether European medical students can prescribe rationally and safely. Eur J Clin Pharmacol.

[344] Chin P.K.L., Charles K., Murnion B. (2023). Evaluation of the Prescribing Skills Assessment implementation, performance and medical student experience in Australia and New Zealand. Br J Clin Pharmacol.

[345] Harrison C., Hilmer S. (2019). The prescribing skills assessment: a step towards safer prescribing. Aust Prescr.

[346] Wallerstedt S.M., Wallerstedt M., Wallerstedt S. (2013). The specialty clinical pharmacology needs to be examined separately to guarantee a sufficient level of knowledge in medical students. Eur J Clin Pharmacol.

[347] Hauser K., Matthes J. (2017). Medical students’ medication communication skills regarding drug prescription-a qualitative analysis of simulated physician-patient consultations. Eur J Clin Pharmacol.

[348] Blackmore A., Kasfiki E.V., Purva M. (2018). Simulation-based education to improve communication skills: a systematic review and identification of current best practice. BMJ Simul Technol Enhanc Learn.

[349] Turing A.M. (1950). Computing machinery and intelligence. Mind.

[350] OpenAI Introducing ChatGPT. https://openai.com/index/chatgpt/.

[351] Biever C. (2023). ChatGPT broke the Turing test-the race is on for new ways to assess AI. Nature.

[352] British Pharmacological Society. Pharmacology education and employment landscape report: Webb. Jisc Artificial Intelligence. A generative AI primer. Accessed April 1, 2026. https://nationalcentreforai.jiscinvolve.org/wp/2026/01/27/generative-ai-primer/

[353] Reich R. Guardian news. Now AI can write students’ essays for them, will everyone become a cheat? Accessed April 1, 2026. https://www.theguardian.com/commentisfree/2022/nov/28/ai-students-essays-cheat-teachers-plagiarism-tech

[354] World Economic Forum Jobs of tomorrow: large language models and jobs - A business toolkit. https://www3.weforum.org/docs/WEF_Jobs_of_Tomorrow_Large_Language_Models_and_Jobs_2023.pdf.

[355] Kleinman Z. Why Google's 'woke' AI problem won't be an easy fix. https://www.bbc.co.uk/news/technology-68412620.

[356] Dave T., Athaluri S.A., Singh S. (2023). ChatGPT in medicine: an overview of its applications, advantages, limitations, future prospects, and ethical considerations. Front Artif Intell.

[357] Gurman M. Samsung bans staff’s AI use after spotting ChatGPT data leak. https://www.bloomberg.com/news/articles/2023-05-02/samsung-bans-chatgpt-and-other-generative-ai-use-by-staff-after-leak.

[358] Morreale F., Bahmanteymouri E., Burmester B., Chen A., Thorp M. (2023). The unwitting labourer: extracting humanness in AI training. AI Soc.

[359] Berthelot A., Caron E., Jay M., Lefèvre L. (2024). Estimating the environmental impact of Generative-AI services using an LCA-based methodology. Procedia CIRP.

[360] Katz D.M., Bommarito M.J., Gao S., Arredondo P. (2024). GPT-4 passes the bar exam. Philos Trans A Math Phys Eng Sci.

[361] Kung T.H., Cheatham M., Medenilla A. (2023). Performance of ChatGPT on USMLE: potential for AI-assisted medical education using large language models. PLoS Digit Health.

[362] Ibrahim H., Liu F., Asim R. (2023). Perception, performance, and detectability of conversational artificial intelligence across 32 university courses. Sci Rep.

[363] Scarfe P., Watcham K., Clarke A., Roesch E. (2024). A real-world test of artificial intelligence infiltration of a university examinations system: a “Turing Test” case study. PLoS One.

[364] Sadasivan V.S., Kumar A., Balasubramanian S., Wang W., Feizi S. (Preprint. Posted online March 17, 2023). Can AI-generated text be reliably detected?. arXiv.

[365] OpenAI New AI classifier for indicating AI-written text. https://openai.com/index/new-ai-classifier-for-indicating-ai-written-text/.

[366] Chechitelli A. AI writing detection update from Turnitin’s chief product officer. https://www.turnitin.com/blog/ai-writing-detection-update-from-turnitins-chief-product-officer.

[367] Ghosal S.S., Chakraborty S., Geiping J., Huang F., Manocha D., Bedi A.S. (Preprint. Posted online October 23, 2023). Towards possibilities & impossibilities of Ai-generated text detection: a survey. arXiv.

[368] Liang W., Yuksekgonul M., Mao Y., Wu E., Zou J. (2023). GPT detectors are biased against non-native English writers. Patterns.

[369] Hamilton A. (2024). Artificial intelligence and healthcare simulation: the shifting landscape of medical education. Cureus.

[370] Scherr R., Halaseh F.F., Spina A., Andalib S., Rivera R. (2023). ChatGPT interactive medical simulations for early clinical education: case study. JMIR Med Educ.

[371] Webb J.J. (2023). Proof of concept: using ChatGPT to teach emergency physicians how to break bad news. Cureus.

[372] Skryd A., Lawrence K. (2024). ChatGPT as a tool for medical education and clinical decision-making on the wards: case study. JMIR Form Res.

[373] Siu A.H.Y., Gibson D., Mu X. (2023). Employing large language models for surgical education: an in-depth analysis of ChatGPT-4. J Med Educ.

[374] Cheung B.H.H., Lau G.K.K., Wong G.T.C. (2023). ChatGPT versus human in generating medical graduate exam multiple choice questions-A multinational prospective study (Hong Kong S.A.R., Singapore, Ireland, and the United Kingdom). PLoS One.

[375] Laupichler M.C., Rother J.F., Grunwald Kadow I.C., Ahmadi S., Raupach T. (2024). Large language models in medical education: comparing ChatGPT- to human-generated exam questions. Acad Med.

[376] Morjaria L., Burns L., Bracken K. (2024). Examining the efficacy of ChatGPT in marking short-answer assessments in an undergraduate medical program. IME.

[377] Schultze T., Kumar V.S., McKeown G., O’Connor P.A., Rychlowska M., Sparemblek K. Using Large Langue Models to Augment (Rather Than Replace) Human Feedback in Higher Education Improves Perceived Feedback Quality.

[378] Pan S.C., Zung I., Imundo M.N., Zhang X., Qiu Y. (2023). User-generated digital flashcards yield better learning than premade flashcards. J Appl Res Mem Cogn.

[379] Kasneci E., Sessler K., Küchemann S. (2023). ChatGPT for good? On opportunities and challenges of large language models for education. Learn Individ Differ.

[380] Lakshan M.T.D., Chandratilake M., Drahaman A.M.P., Perera M.B. (2024). Exploring the pros and cons of integrating artificial intelligence and ChatGPT in medical education: a comprehensive analysis. Ceylon J Otolaryngol.

[381] Breeding T., Martinez B., Patel H. (2024). The utilization of ChatGPT in reshaping future medical education and learning perspectives: a curse or a blessing?. Am Surg.

[382] Araji T., Brooks A.D. (2024). Evaluating the role of ChatGPT as a study aid in medical education in surgery. J Surg Educ.

[383] Rong G., Mendez A., Bou Assi E., Zhao B., Sawan M. (2020). Artificial intelligence in healthcare: review and prediction case studies. Engineering.

[384] Iqbal J., Cortés Jaimes D.C., Makineni P. (2023). Reimagining healthcare: unleashing the power of artificial intelligence in medicine. Cureus.

[385] Ryan D.K., Maclean R.H., Balston A., Scourfield A., Shah A.D., Ross J. (2024). Artificial intelligence and machine learning for clinical pharmacology. Br J Clin Pharmacol.

[386] Marques L., Costa B., Pereira M. (2024). Advancing precision medicine: a review of innovative in silico approaches for drug development, Clinical Pharmacology and personalized healthcare. Pharmaceutics.

[387] van der Lee M., Swen J.J. (2023). Artificial intelligence in pharmacology research and practice. Clin Transl Sci.

[388] M Bran A.M., Cox S., Schilter O., Baldassari C., White A.D., Schwaller P. (2024). Augmenting large language models with chemistry tools. Nat Mach Intell.

